# Unlabeled aspirin as an activatable theranostic MRI agent for breast cancer

**DOI:** 10.7150/thno.53147

**Published:** 2022-01-24

**Authors:** KowsalyaDevi Pavuluri, Ethan Yang, Vinay Ayyappan, Kanchan Sonkar, Zheqiong Tan, Caitlin M. Tressler, Shaowei Bo, Adnan Bibic, Kristine Glunde, Michael T McMahon

**Affiliations:** 1 Division of MR Research, The Russell H. Morgan Department of Radiology and Radiological Science; The Johns Hopkins University School of Medicine, Baltimore, MD.; 2 Division of Cancer Imaging Research, The Russell H. Morgan Department of Radiology and Radiological Science; The Johns Hopkins University School of Medicine, Baltimore, MD.; 3 The Department of Biological Chemistry, The Johns Hopkins University School of Medicine, Baltimore, MD.; 4 Sidney Kimmel Comprehensive Cancer Center, The Johns Hopkins University School of Medicine, Baltimore, MD.; 5 F.M. Kirby Research Center for Functional Brain Imaging, Kennedy Krieger Institute, Baltimore, MD.; 6 Department of Medical Laboratory, The Central Hospital of Wuhan, Tongji Medical College, Huazhong University of Science and Technology, Wuhan, Hubei, China

**Keywords:** CEST, MRI, aspirin, salicylic acid, breast cancer, gadolinium

## Abstract

**Rationale:** Chemical exchange saturation transfer (CEST) magnetic resonance imaging (MRI) is emerging as an alternative to gadolinium-based contrast MRI. We have evaluated the possibility of CEST MRI of orthotopic breast tumor xenografts with unlabeled aspirin's conversion to salicylic acid (SA) through various enzymatic activities, most notably inhibition of cyclooxygenase (COX)-1/-2 enzymes.

**Methods**: We measured the COX-1/-2 expression in four breast cancer cell lines by Western Blot analysis and selected the highest and lowest expressing cell lines. We then performed CEST MRI following aspirin treatment to detect SA levels and ELISA to measure levels of downstream prostaglandin E2 (PGE2). We also injected aspirin into the tail vein of mice growing orthotopic tumor xenografts which expressed high and low COX-1/-2 and acquired SA CEST MR images of these tumor xenografts for up to 70 minutes. Tumors were then harvested to perform Western Blot and ELISA experiments to measure COX-1/-2 expression and PGE2 levels, respectively.

**Results**: Western Blots determined that SUM159 cells contained significantly higher COX-1/-2 expression levels than MDA-MB-231 cells, in line with higher levels of downstream PGE2. SA CEST MRI yielded similar contrast at approximately 3% for both cell lines, independent of COX-1/-2 expression level. PGE2 levels decreased by about 50% following aspirin treatment. Results from our mouse study aligned with cultured cells, the overall SA CEST MRI contrast in both MDA-MB-231 and SUM159 tumor xenograft models was 5~8% at one hour post injection. PGE2 levels were ten times higher in SUM159 than MDA-MB-231 and decreased by 50%. The CEST contrast directly depended on the injected dose, with ~6%, ~3% and ~1.5% contrast observed following injection of 100 µL of 300 mM, 200 mM and 150 mM aspirin, respectively.

**Conclusions**: Our data demonstrate the feasibility of using aspirin as a noninvasive activatable CEST MRI contrast agent for breast tumor detection.

## Introduction

Breast cancer remains a highly prevalent global disease, with nearly 2.3 million new diagnoses and more than 600,000 deaths in 2020 alone, making it the leading cause of cancer death worldwide 1. The standard-of-care diagnostic screenings rely on x-ray mammography 2, except for high-risk women who are monitored by magnetic resonance imaging (MRI) with gadolinium-containing contrast agents administered to highlight breast tumors in T1-weighted MRI through alterations in tumor perfusion 3. This method produces high-resolution images with exquisite soft tissue contrast and has long been recognized as an outstanding tool for characterizing breast cancer and other malignancies 4-6. However, there are concerns to apply this to patients with poor kidney function 7, 8, and several studies have pointed to the bioaccumulation of gadolinium-containing MRI contrast agents after years of injection on an annual basis, including in children 9-11, raising concern about their long-term effects.

Chemical Exchange Saturation Transfer (CEST) MRI has emerged as a possible alternative to gadolinium contrast MRI. This important technology for molecular imaging indirectly detects low concentrations of compounds with suitable labile protons through the application of frequency selective radiofrequency (RF) saturation pulses on these protons. These RF saturation pulses result in a net loss in water longitudinal magnetization through transfer of saturation by chemical exchange 12-15. The chemical shift dependence is an important feature of CEST MRI, allowing discrimination among different agents through what has been described as multi-color 16-18 or multi-frequency CEST MRI 14, 19. To date, glucose (and analogs) CEST imaging has shown the most promise for tumor detection based on testing this technology on animal models and patients in clinical scanners 20-22. Similarly, we have recently discovered several salicylates that possess favorable properties for CEST MRI, allowing highly amplified and specific detection of these compounds 23-25. Of them, salicylic acid (SA) proved to be the most promising because of its large proton shift from water at 9.6 ppm and its suitable exchange rate, making its signal easily discernible among other metabolites 23.

SA is an attractive CEST contrast agent owing to the potency of its parent metabolite, acetyl salicylic acid (ASA), better known as aspirin 26, 27. A popular non-steroidal anti-inflammatory drug (NSAID) 28, aspirin is the most widely used drug on the market with 44,000 tons consumed globally per year 29. Beyond its usefulness in pain management, several epidemiological studies have associated its use with chemoprevention from colorectal, breast and pancreatic cancers 30-36, including a 41% reduction for the risk of developing luminal A breast cancer associated with lifetime use of aspirin 31, 37. The therapeutic action of aspirin results from its irreversible inhibition of cyclooxygenase (COX)-1 and COX-2 through the acetylation of the serine residue in the catalytic site, which in turn lowers prostaglandin synthesis and reduces inflammation, pain, fever, blood flow, vascular permeability, and immune activation 38-41. This mechanism converts aspirin to SA, providing a direct link between aspirin metabolism and CEST signal.

Several previous studies have explored the possibility of NSAIDs for MRI. For example, Kim *et al.* directly labelled NSAIDs with gadolinium chelates to detect their accumulation with standard T1 weighted imaging 42. While promising, this method suffers from the same limitations as other gadolinium-based techniques. In another example, Zacharias *et al*. used ^13^C-labeled aspirin and hyperpolarization to follow its biodistribution and metabolism with magnetic resonance spectroscopy for a short time period of a few seconds until the hyperpolarized signal had decayed 43. The short timespan makes this method challenging to implement on clinical scanners or provide spectroscopic imaging beyond minutes. CEST MRI with unlabeled aspirin overcomes the toxicity from gadolinium-based MRI and time limitations of hyperpolarization. In this study, we examined the possibility of injecting aspirin as an activable theranostic MRI agent for breast cancer. Specifically, we sought to study the effects of COX-1 and COX-2 expression levels on SA CEST contrast and prostaglandin E2 (PGE2) production. Our results showed strong and similar CEST MRI contrast for triple negative breast cancer cells *in vitro* and orthotopic breast tumor xenografts in mice *in vivo*, regardless of their COX-1/-2 expression levels, while PGE2 significantly decreased in the high COX-1/-2 model. The robustness of the SA CEST MRI contrast makes this novel method a viable contender for future cancer diagnostic applications.

## Results

**Figure 1:**
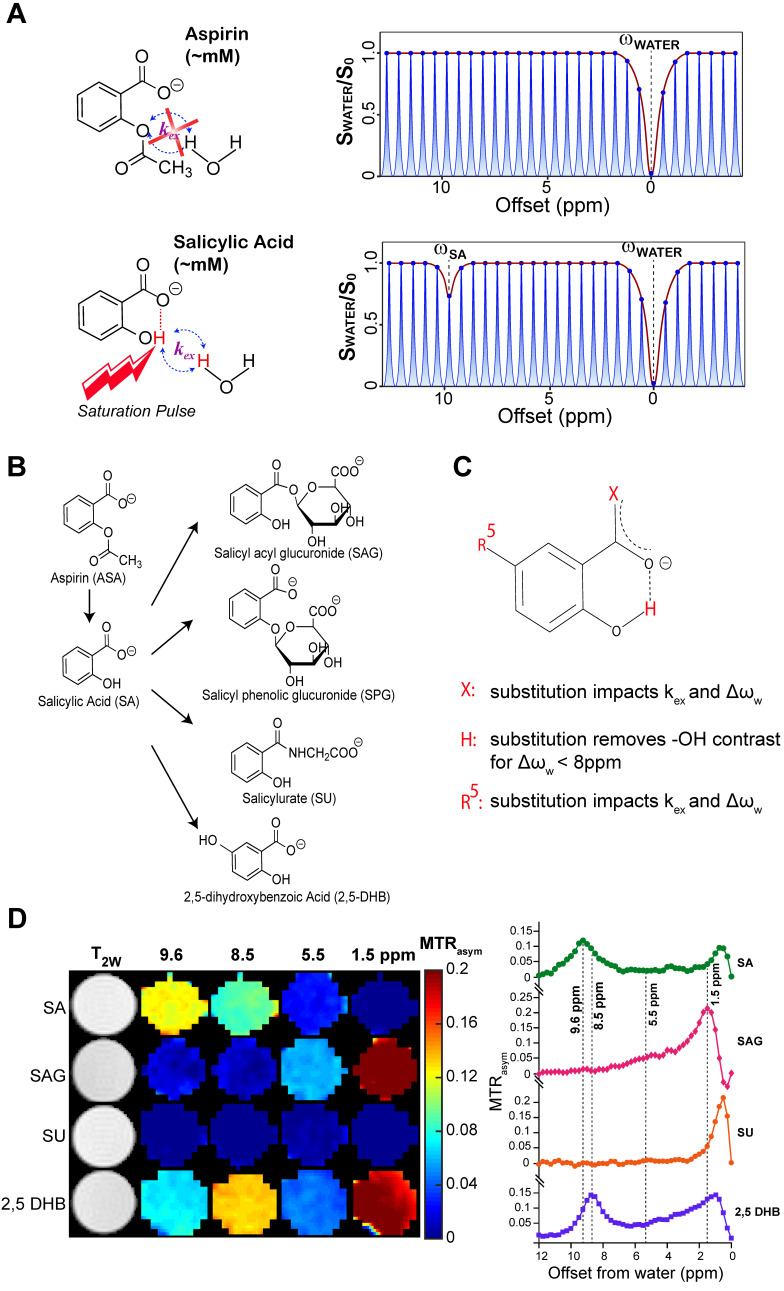
** CEST-MRI detection of aspirin metabolites. (A)** Schematic depicting that salicylic acid, unlike aspirin, can be detected by CEST MR due to the presence of an exchangeable proton that exchanges with bulk water. **(B)** Molecular structures of salicylate compounds along the metabolic pathway of aspirin. While SAG, SPG, and SU are major metabolites of aspirin, 2,5-DHB is a minor metabolite. **(C)** List of rules showing the effect of various substitutions on exchange rates and the offset frequencies at which exchangeable protons resonate for these salicylate compounds. **(D)** At the left are multi-color CEST MTR_asym_ images corresponding to the main frequencies allowing detection of the salicylate compounds SA, SAG, SU, and 2,5 DHB. As shown, at pH 6.6, SA produces the strongest contrast at a relatively isolated frequency of ~9.6 ppm. Although there is a small overlap with the peak produced by 2,5-DHB, the latter is a minor metabolite and not detected in remarkable quantities.

The salicylates are particularly well suited for detection by CEST MRI owing to the presence of an intramolecular hydrogen bond between the carboxylate anion at position 1 on the phenyl ring and a labile hydroxyl proton at the adjacent position 2 which is not present in aspirin (**Figure 1A**). We have previously discovered that replacements of this carboxyl group could remove the contrast at ~9.6 ppm 14, 15. The metabolic pathway for aspirin and SA is depicted in **Figure 1B**. Downstream aspirin metabolites display perturbed hydrogen bonding arrangements through deacetylation, or addition of glucuronic acid, glycine or hydroxyl groups 44-46. We first sought to isolate these metabolites in phantoms based on their CEST MR spectra at various saturation powers. In humans, three conjugates of salicylate are formed directly: the main metabolite salicylurate (SU), and two glucuronides, i.e. salicyl acyl glucuronide (SAG) and salicyl phenolic glucuronide (SPG) 47.

As seen from **Table S1** and **Figure 1D**, the free -OH group in SA produces CEST contrast, while SAG and SU do not display CEST contrast at ~9.6 ppm. Because SPG does not contain an -OH group at position 2 (**Figure 1B**), there is no signal for this metabolite at 9.6 ppm either 24. Contrast is retained but shifted to 8.5 ppm for 2,5-dihydroxybenzoic acid (2,5-DHB), which is a minor metabolite, constituting less than 5% of eliminated drug 47. We analyzed our CEST MR data to measure the exchange rates (*k_ex_*) of the main SA metabolites at a range of pH values between 6.3 and 7.2 (**Table S1**). CEST MTR_asym_ spectra of these metabolites for pH values between 6.3 and 7.2 at RF saturation power (B_1_) = 6 μΤ are shown in **Figure S1**. We have also determined that the free -OH group in SA retains its contrast in blood serum (**Figure S2**). We have thus identified conditions that isolate SA from other metabolites when the saturation pulse is placed at 9.6 ppm, with aspirin (ASA) and SPG not being detected at all by CEST MRI due to the absence of a hydroxyl group.

**Figure 2:**
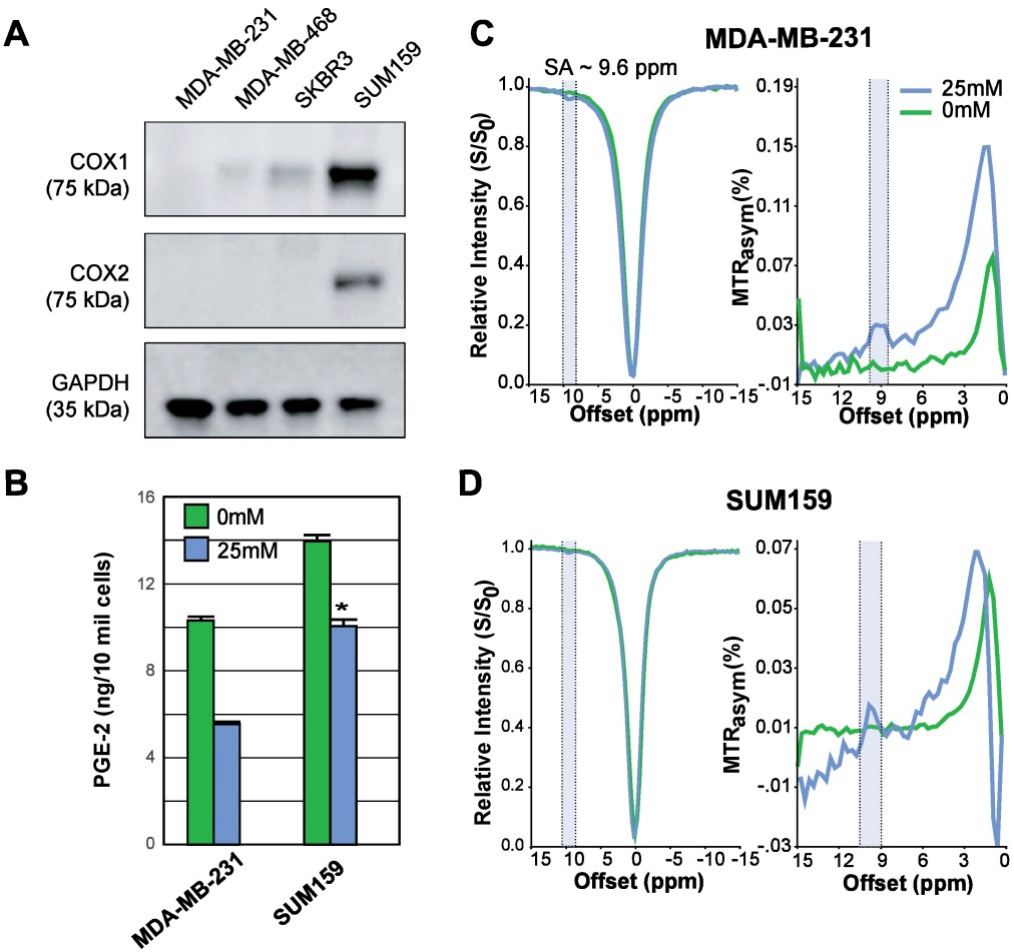
** COX-1/-2 expression, PGE2 assay, and CEST MR detection of SA in triple negative breast cancer cell lines. (A)** Western blot analysis for COX-1 and COX-2 protein expression in MDA-MB-231, MDA-MB-468, SKBR3 and SUM159 breast cancer cell lines without aspirin treatment. MDA-MB-231 has the lowest, while SUM159 has the highest COX-1 and COX-2 expression level.** (B)** Comparison of PGE2 levels in MDA-MB-231 and SUM159 cells treated with 0 mM or 25 mM aspirin. In both cell lines, there is a significant drop in PGE2 concentration following treatment. Data shown are mean ± standard deviation. * represents p<0.5. **(C)** MDA-MB-231 and **(D)** SUM159 z-spectra and MTR_asym_ plots showing SA production after 20 mins in 0 mM or 25 mM of DL-Lys aspirin. Signal generated by SA was visible at ~9.6ppm for both cell lines following treatment with 25 mM of aspirin.

Aspirin permanently inhibits COX-1/COX-2 through serine acetylation in the active site, producing SA as a result 26. Given that COX-1 and -2 enzymes catalyze the rate-limiting step of prostaglandin synthesis and that the COX-PGE2 pathway in particular promotes carcinogenesis and tumor progression 48, 49, we hypothesized that noninvasive CEST-MRI monitoring of SA accumulation in cancer cells and tumors could provide a readout of COX-PGE2 pathway blockage. To test this hypothesis, we first determined the COX-1/-2 expression levels of four candidate breast cancer cell lines, MDA-MB-231, MDA-MB-468, SKBR3 and SUM159, by gel electrophoresis and Western blotting. From our results (**Figure 2A**), SUM159 displayed the highest COX-1 level, while MDA-MB-231 had the lowest COX-1 expression. SUM159 was the only cell line with detectable COX-2 expression. Consequently, we selected SUM159 as our high COX model and MDA-MB-231 as our low COX model for further experimentation. An ELISA PGE2 assay of these cells revealed an approximately 50% higher PGE2 level in the SUM159 cell line compared to the MDA-MB-231 cell line (**Figure 2B**), which was 14.08 ± 1.04 ng/10 million cells compared to 10.75 ± 0.38 ng/10 million cells for SUM159 and MDA-MB-231 cells, respectively. Treatment of both cell lines with 25 mM aspirin for 20 minutes led to an approximate 50% drop in PGE2 levels to 10.16 +/- 1.28 ng/10 million cells and 5.71 +/- 0.26 ng/10 million cells for SUM159 and MDA-MB-231 cells, respectively. Finally, we measured the CEST MR signal to detect SA buildup by measuring MTR_asym_ at ~9.6 ppm. Despite the significant differences in COX-1/-2 expression and PGE2 levels, the overall SA CEST MRI signal was similar in the two cell lines, with MDA-MB-231 cells at an average of 3% (**Figure 2C**) and SUM159 at an average of 2% (**Figure 2D**). This suggests that the COX-PGE2 pathway is not solely responsible for converting aspirin to SA and thereby generating the CEST MR signal.

**Figure 3:**
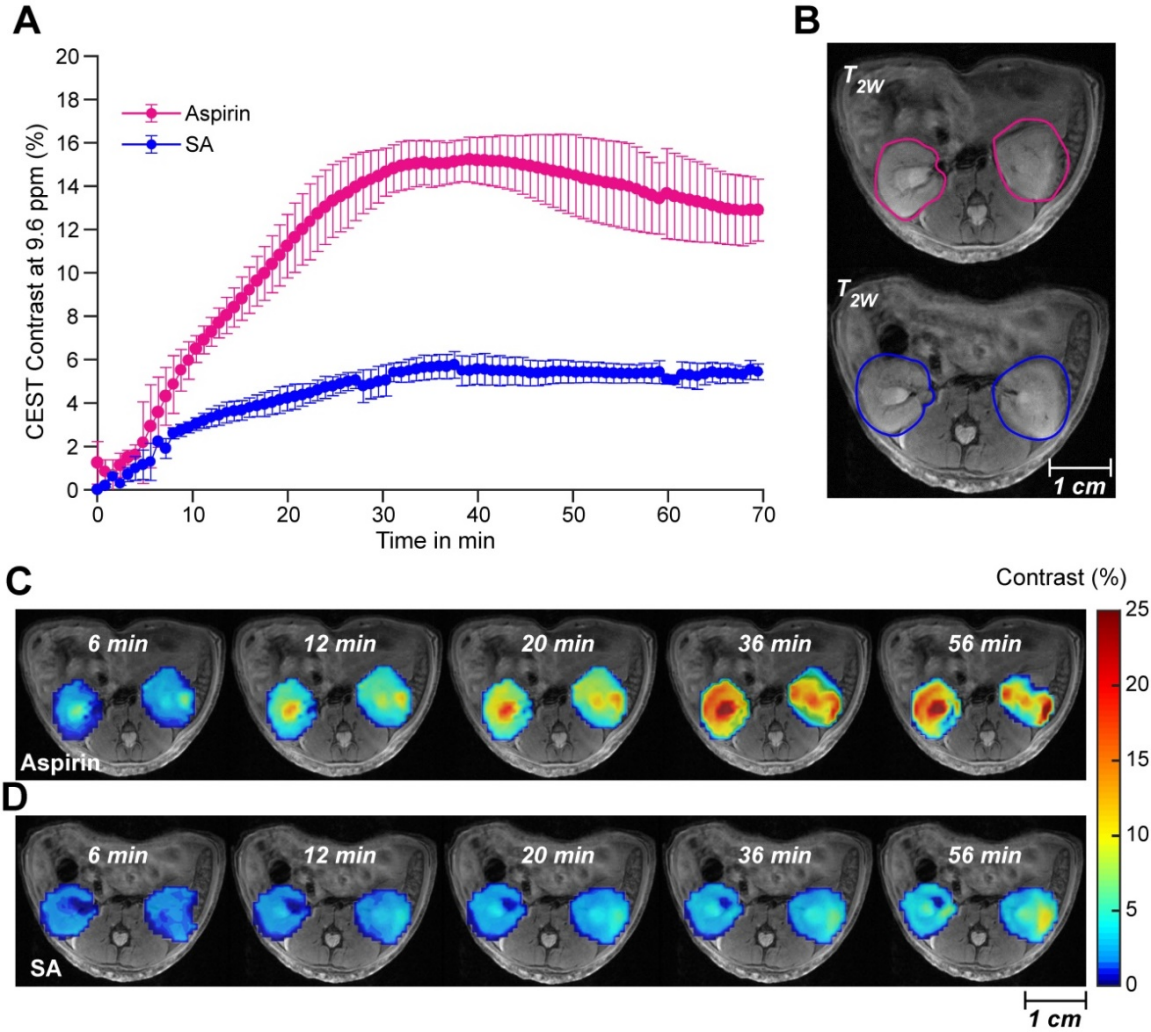
** Renal CEST-MRI contrast following aspirin or SA injection. (A)** Average renal contrast buildup over time at 9.6 ppm for aspirin and SA in mice (n=3 each) at saturation B_1_ = 6 μΤ. **(B)** High-resolution T_2w_ images with the ROIs drawn over the two kidneys for CEST MRI contrast measurement; **(C)** Dynamic CEST MRI contrast images calculated within one hour of aspirin and SA administration. Contrast images were calculated for an ROI selected on both kidneys and overlaid on a high-resolution T_2w_ image.

We proceeded to further evaluate aspirin as an activatable contrast agent for detecting SA CEST MRI *in vivo*. First, we assessed the feasibility of detecting SA buildup *in vivo* by monitoring the clearance of SA and aspirin through the kidneys in healthy mice. We injected 100 µL of 300 mM solutions of either injectable aspirin (DL-Lys aspirin) or sodium salicylate and collected CEST MR images of both kidneys, which are known to be the main clearance route for aspirin and its metabolites. While direct injections of sodium salicylate led to approximately 5% CEST MRI contrast throughout the kidneys, injection of DL-Lys aspirin resulted in an about 3-fold higher contrast (~15%) as shown in **Figure 3A**. Dynamic SA CEST MRI contrast maps were obtained with a saturation pulse at 9.6 ppm at various time points following administration of DL-Lys aspirin or SA and are displayed in **Figure 3C-D**. At 6 min following administration, <2% CEST MRI contrast was observed for both aspirin and SA. After 6 min, the renal contrast uptake rate was faster for DL-Lys aspirin compared to that of SA, with both peaking at 30-40 min following administration. At this time point, the CEST MRI contrast was 3 times higher for aspirin compared to that of SA. These observations demonstrate that deacetylation of injectable aspirin generates larger SA CEST MRI contrast than direct injection of SA.

**Figure 4:**
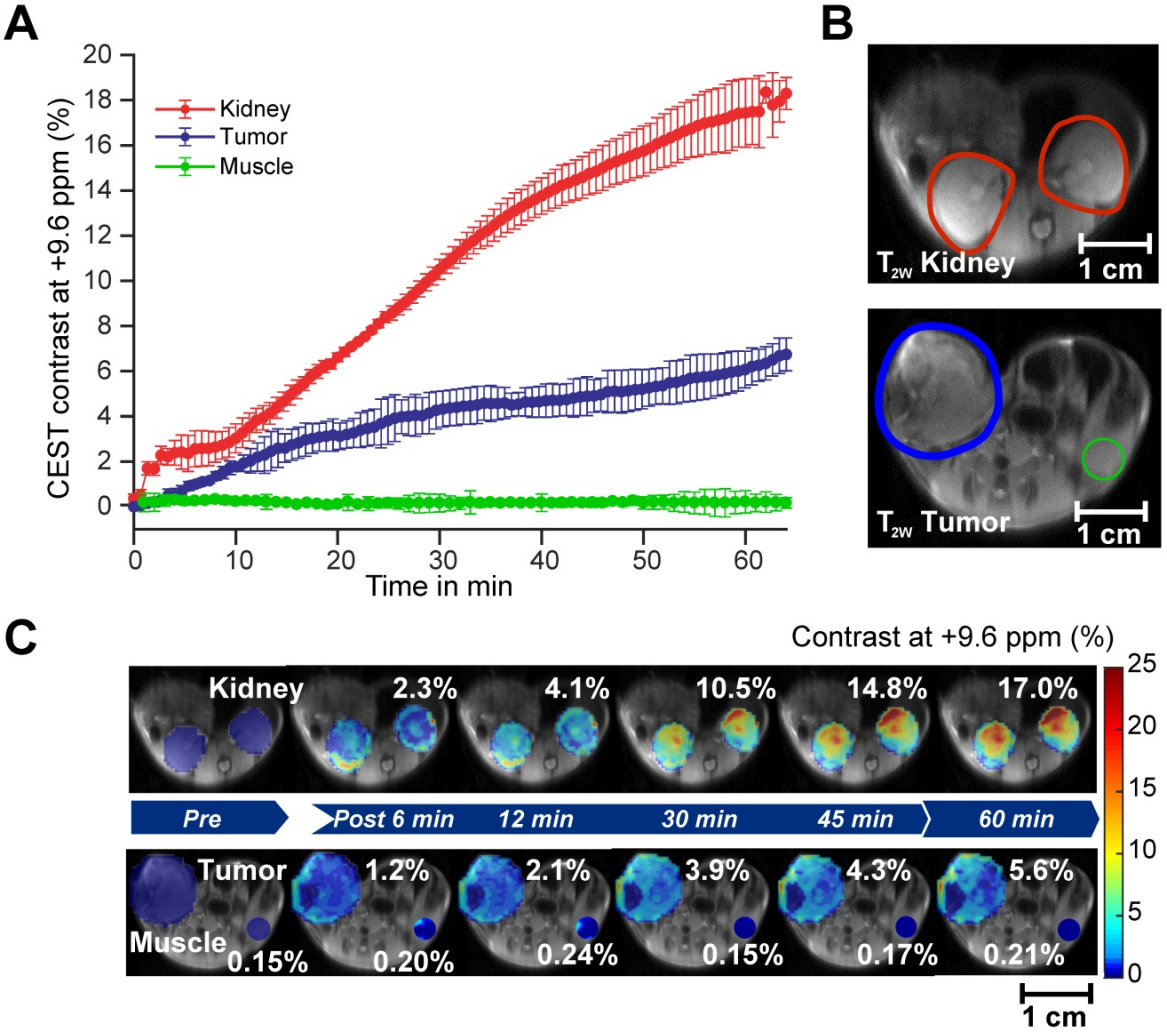
** CEST MRI detection of salicylic acid buildup in triple-negative MDA-MB-231 breast tumor xenografts in the mammary fat pad of mice following aspirin injection. (A)** Time course of CEST MRI contrast agent buildup in kidney (red), tumor (blue), and muscle (green); **(B)** High-resolution T_2w_ images marked with kidney (red), tumor (blue) and muscle (green) ROI outlines; **(C)** Dynamic CEST MRI contrast images of pre- and post-injection of 300 mM aspirin from kidney and tumor and muscle overlaid on T_2w_ images along with the average CEST MRI contrast at 9.6 ppm as indicated. CEST MRI contrast increases continuously over the course of the one-hour imaging period.

**Figure 5:**
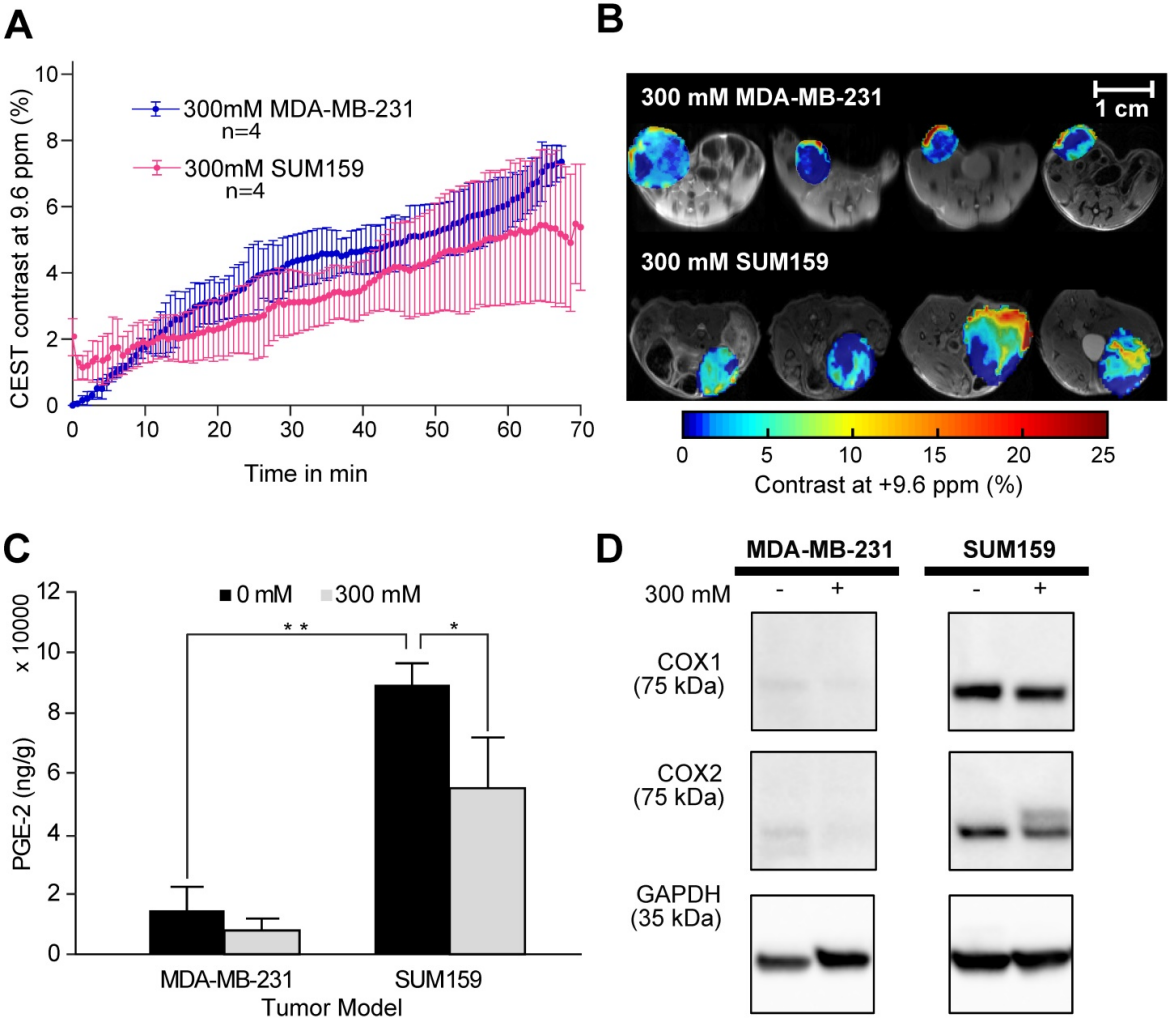
** CEST MRI, PGE2 and COX-1/-2 comparison of MDA-MB-231 and SUM159 tumor xenografts following aspirin treatment. (A)** Time course of CEST MRI contrast buildup in the tumor models after injection. There is no significant difference in CEST MRI signal between the two tumor models. **(B)** Post-injection dynamic CEST MR contrast images of tumors overlaid on T_2w_ images along with the average CEST contrast at 9.6 ppm as indicated. **(C)** PGE2 assay of the tumor xenografts with and without aspirin treatment. SUM159 contained significantly higher levels of PGE2 compared to MDA-MB-231, regardless of aspirin treatment. Aspirin treatment led to significant decrease in PGE2 in both tumor models compared to corresponding untreated controls. Data shown are mean ± standard deviation. * represents p<0.5, ** represents p<0.01. **(D)** Representative Western blots of COX-1 and COX-2 expression levels of tumor xenografts from mice treated with and without 300 mM DL-Lys aspirin. SUM159 expressed significantly higher COX-1 and COX-2 levels compared to MDA-MB-231. Aspirin treatment did not significantly affect COX-1 and COX-2 expression levels. Supplementary Figure 7 contains the Western blots from all biological repeats.

After confirming the SA CEST MRI contrast in healthy mouse kidneys, we applied this approach in the low COX-1/-2 MDA-MB-231 (n=4) and high COX-1/-2 SUM159 (n=4) breast tumor xenograft model in mice. In MDA-MB-231 tumor bearing mice, sixty minutes after injecting the mice with 100 µL of 300 mM DL-Lys aspirin, we observed about 1/3 of SA CEST MRI contrast buildup in tumor compared to that of kidney, with control muscle tissue displaying no change in contrast (**Figure 4A-C**). CEST MR images acquired from kidney and tumor of representative mice pre- and post-aspirin injection are shown without masking in **Figure S3**. Compared to the low COX-1 and COX-2 model MDA-MB-231, the SUM159 tumor xenografts (n=4) contained slightly elevated levels of SA initially after DL-Lys aspirin injection, which continued to build up to comparable contrast levels in both tumor xenograft models at around 6% irrespective of their COX-1/-2 expression level (**Figure 5A**). CEST MRI images acquired at 60 minutes post aspirin injection without tumor mask and dynamic SA CEST MRI images of all biological replicates for both SUM159 and MDA-MB-231 tumor xenografts are presented in **Figures S4 and S5**, respectively. The overall enhancement pattern in the two orthotopic tumor xenograft models was similar (**Figure 5B**). In some cases, we observed elevated SA CEST MRI contrast near the tumor rim, most likely representing vascularized, metabolically active cells in the rim of tumors with respect to a hypoxic or necrotic tumor core 50. We compared gadolinium enhanced MRI (Gadoteridol) with SA CEST MRI results in three MDA-MB-231 and three SUM159 mice, where gadolinium enhanced MRI was acquired 2 days prior to their CEST MRI acquisition to ensure complete clearance of gadolinium-containing contrast agent prior to aspirin injection. These comparative measurements revealed contrast enhancement in both breast tumor xenograft models with both types of contrast enhanced MRI (**Figure S6**). Intra-tumoral PGE2 levels of the two tumor models were significantly different. SUM159 tumor xenografts contained a more than five-fold increase in PGE2 compared to MDA-MB-231 tumor xenografts (**Figure 5C**), measuring 89.2 ± 7.1 µg/g and 54.9 ± 16.8 µg/g in SUM159 tumors treated with 0 mM and 300 mM aspirin, respectively, *versus* 14.5 ± 7.8 µg/g and 7.9 ± 2.7 µg/g for the MDA-MB-231 tumor xenografts. Western blot analysis of the tumor xenograft protein lysates consistently showed much higher COX-1/-2 levels in SUM159 tumor xenografts compared to MDA-MB-231 tumor xenografts (**Figure 5D**). While PGE2 levels did decrease as a result of aspirin treatment in both tumor models (**Figure 5C**), Western blot data did not indicate a significant difference in COX-1/-2 expression level following treatment (**Figure 5D, Figure S7**).

**Figure 6:**
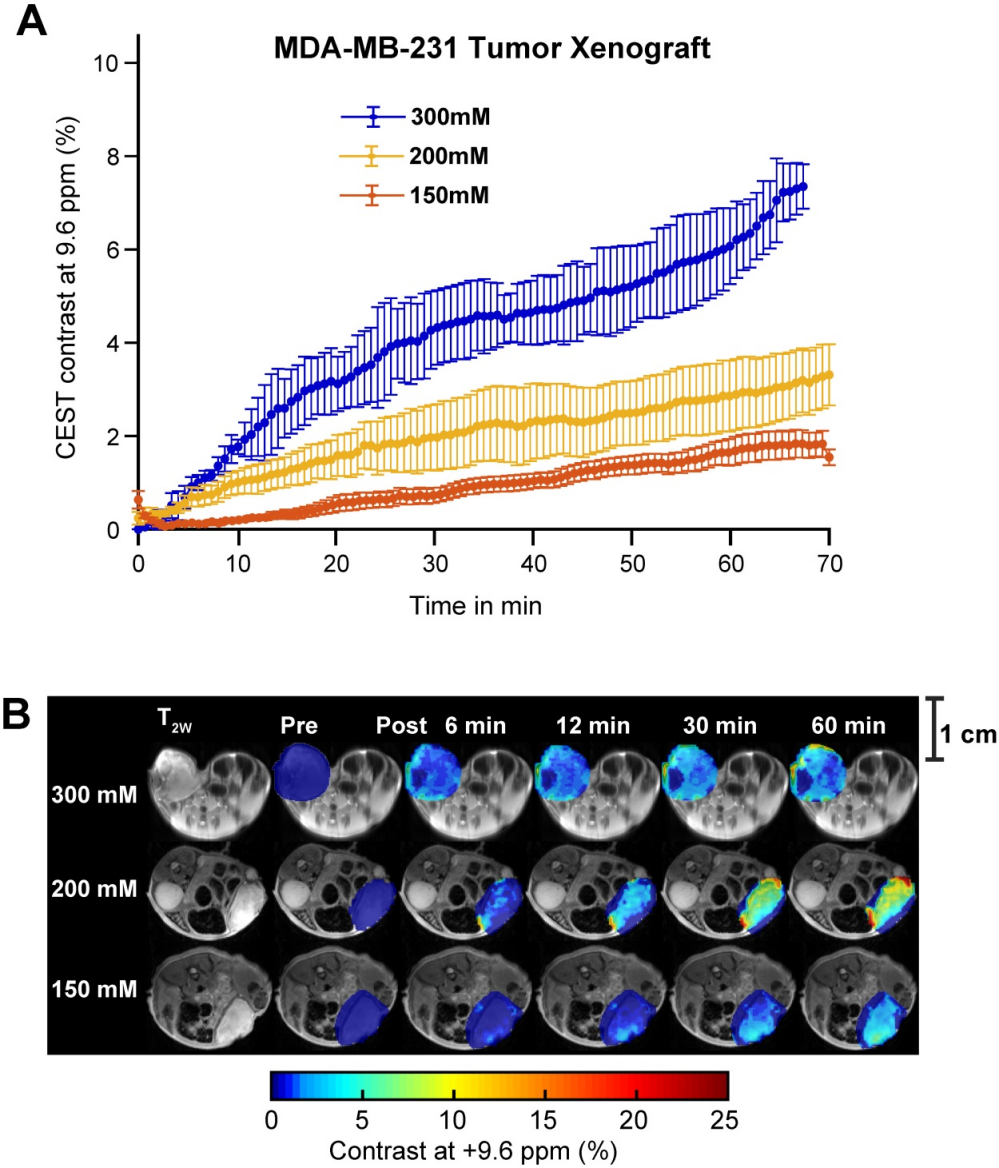
** Aspirin concentration effects on overall CEST MRI contrast in MDA-MB-231 tumor xenograft. (A)** Time course of CEST MRI contrast agent buildup in the tumor region following administration of 300 mM (blue), 200 mM (yellow) or 150 mM (red) DL-Lys aspirin through the tail vein. **(B)** High resolution T_2w_ image and pre- and post-injection dynamic CEST MR contrast images of the tumor regions over the course of 60 mins, with the average CEST MRI contrast at 9.6 ppm at each time point as indicated. There is an significant increase in CEST MRI contrast with an increased dose of injected aspirin. Error bars represent one standard deviation. See Supplementary Figures 9, 10 and 11 for data from biological replicates.

While SA CEST MRI signal buildup in tumors was not indicative of COX-1/-2 expression level or COX-PGE2 pathway activity in the tumor, the overall contrast did align with the concentration of the injected aspirin. Intravenous injection of 300 mM of aspirin in mice growing MDA-MB-231 tumor xenografts resulted in 7% CEST MRI contrast at 60 minutes following injection, while mice treated with 200 mM and 150 mM displayed ~3% and ~1.5% tumor CEST MRI contrast at the 60-minute time point, respectively (**Figure 6A**). The overall SA distribution and the resulting CEST MRI enhancement pattern was similar for the three different injected doses of aspirin (**Figure 6B, Figure S5, S8 and S9**). The PGE2 levels consistently decreased with increasing aspirin dose, which was not significant in the low COX-1/-2 model of MDA-MB-231 tumor xenografts (**Figure S10**). Finally, we orthogonally confirmed the observed aspirin metabolite levels in SUM159 tumor xenografts by using matrix-assisted laser desorption/ionization (MALDI) time of flight (TOF) mass spectrometry tissue profiling. MALDI-TOF showed that in SUM159 tumors, the only detected aspirin metabolite was SA with no detection of 2,5-DHB in these spectra as compared to MALDI-TOF mass spectra of pure standard compounds and matrix background (**Figure S11**).

## Discussion

In this study, we have demonstrated the use of commercially available aspirin as an activatable theranostic CEST agent for breast cancer imaging and have developed an innovative CEST MR imaging strategy for monitoring aspirin metabolism. We determined the feasibility of detecting SA CEST MRI contrast at ~9.6 ppm following aspirin addition to human breast cancer cells and set up optimized protocols for *in vivo* CEST MRI contrast monitoring of SA after intravenously administering aspirin. Systemic administration of aspirin generated up to 6% CEST MRI contrast in orthotopic triple-negative MDA-MB-231 and SUM159 breast tumor xenografts. This level of contrast was, on average, three times greater than that from directly injecting SA in the form of sodium salicylate. The higher contrast seen for aspirin injections than SA could be due to several factors including SA having a much greater affinity for albumin than aspirin and the differences in metabolic stability, pKa and lipid solubility which have been described previously 51**.** Also, in this study, we did not take into account differences in T1 as part of the analysis. T1 changes between different tissues or samples can have an impact on the amount of CEST contrast. Bloch simulations have shown that 20% reduction in T1 reduce the CEST contrast by 1% 52. Since aspirin has low toxicity, is used in cancer chemoprevention 53, and readily available worldwide, CEST MRI with intravenous aspirin injection may be a viable dynamic contrast-enhanced imaging approach for detecting breast cancer with theranostic effects. The tumor CEST MRI contrast detected following aspirin injection was specific to SA, as confirmed by MALDI-TOF analyses of treated *versus* untreated tumor tissues from our study. These MALDI-TOF experiments ruled out any presence of 2,5-DHB in the tumor tissues from aspirin-injected mice, which would generate CEST MRI signal close by at ~8. 5 ppm if it were present.

Since aspirin is a well-known inhibitor of COX-1 and COX-2, both of which deacetylate aspirin to form SA, we tested if the amount of *in vivo* SA CEST MRI contrast generated would be affected by COX-1 and COX-2 expression levels of the breast tumor xenograft models used. However, our results revealed no such correlation, as both the low COX-1/-2 model MDA-MB-231 as well as the high COX-1/-2 model SUM159 contained similar levels of *in vivo* SA CEST MRI contrast. These results suggest that most of the injected aspirin was already metabolized to SA prior to arriving at the tumor xenografts. This can be explained by the active hydrolysis of aspirin in the blood and liver by aspirin esterases such as cholinesterases and carboxylesterases in humans, within minutes upon intravenous injection, as well as the presence of COX-1/-2 enzymes in blood serum and liver 27, 54. Despite this, the PGE2 levels in both tumor xenograft models decreased following aspirin injection, particularly for the high COX-1/-2 expressing SUM159 tumor model, aligning with previous studies on the mechanism of action of NSAIDs on prostaglandin production and indicating a therapeutic effect from the aspirin injection that generated *in vivo* SA CEST MRI contrast 26. This observation suggests that the concentration of injected aspirin was at a sufficiently high dose such that enough unbound intact aspirin successfully reached the tumor to produce the inhibitory effect. Based on this proposed mechanism, this new aspirin-enhanced CEST MRI method can be successfully applied to other cancer models with varying levels of COX-1/-2 expression as well 55-57.

Our new approach of SA detection by CEST MRI using aspirin injection for contrast generation could be translated into a powerful tool for cancer imaging with possible future applications in breast, renal, liver and brain imaging. Currently, colorimetric methods 29, 44, and chromatography-based approaches, such as high-pressure liquid chromatography (HPLC) 44, 45, 58, are routine for aspirin quantitation. HPLC is particularly powerful for identifying and quantifying the amount of drug and metabolites in blood or urine. Unfortunately, these chromatography-based methods are limited to the analysis of body fluids and cannot accomplish *in vivo* characterization of drug activity. Moreover, radioactive labeling of aspirin with ^14^C-labeled carboxyl and ^14^C- or ^3^H-labeled acetyl groups and scintillation radiography have been used extensively to understand the pharmacokinetics and inhibitory mechanism of aspirin/SA in both animals and humans 59-61. However, the need for radiochemical synthesis and difficulties with accurate quantification are limitations of scintigraphy-based methods. Given that this SA CEST MRI approach is noninvasive and does not require radiolabeling, it could be used for quantitatively studying and identifying the biodistribution and pharmacokinetics of aspirin and salicylates, with clear applications in cancer detection.

An important question remains on the clinical feasibility of SA CEST MRI. In our current preclinical mouse study, we have observed tumoral SA CEST MRI contrast with as little as 100 µL of 150 mM aspirin administered intravenously, which was roughly 120 mg/kg. This concentration is similar to daily high dose aspirin treatments currently administered in patients: 80-100 mg/kg orally or intravenously in children during the acute stage of Kawasaki disease 62 and ~67 mg/kg orally or intravenously per day in adults for migraine, as determined by the German Migraine and Headache Society and the German Society of Neurology 63-66. For comparison, a typical adult tablet is 325 mg or ~4 mg/kg. It is possible that these high aspirin dosages currently administered in the clinic may be sufficient for SA CEST MRI. In terms of safety, an even higher dose of 143 mg/kg would be required to develop even mild poisoning from orally administered aspirin 67. We also expect that the contrast generated by SA should be lower at 3T than 11.7T based on the reduced saturation powers available for 3T body transmit coils, and this will be the subject of future studies. In our previous human trial we employed D-glucose as a CEST contrast agent, with chemical shifts and exchange rates that are significantly less suitable for CEST imaging than those of SA. Nevertheless for D-glucose, sufficient contrast was observed for detection of uptake on a 7T instrument, with the prior mouse tumor study showing similar contrast as we observed here at 11.7T 68. Furthermore, given that aspirin toxicity in humans will not become severe until more than 140 mg/kg is ingested 67, there remains a wide window for further studies to determine the adequate aspirin dosage for aspirin enhanced CEST MRI for patients in clinical settings.

Like all drugs, aspirin is not without its own safety concerns. While low dosage of aspirin has been attributed to protective effects against numerous ailments including cataracts, cardiovascular diseases and multiple cancers 26, 27, 69, continued oral intake of aspirin at therapeutic doses has been associated with hepatotoxicity and a reversible decline in renal function, with a 10-fold increase in risk attributed to patients with rheumatoid arthritis and children with Reye's syndrome 7, 69. It also leads to increased bleeding events, though rarely have these led to fatal outcomes 69. At high doses employed in our SA CEST MRI study, aspirin has been linked to tinnitus and anemia 69. Despite this, injectable aspirin remains an attractive CEST MRI contrast agent because it does not accumulate in the body like gadolinium-based MRI contrast agents (GBCAs) 7. With an elimination half-life of 90 minutes in patients with normal kidney function, GBCA has been shown by several studies to accumulate in the brain, skin, liver and bone for both linear and chelated gadolinium compounds, possibly leading to erroneous interpretation of future MRI results and eventual toxicity 70. Therefore, we believe that SA CEST MRI through intravenous aspirin administration remains an attractive and promising avenue.

## Methods

***Materials and reagents.*** Unless otherwise noted, all compounds, chemicals and solvents were purchased from Sigma-Aldrich (St. Louis, MO, USA). Solvents were at least HPLC grade, and MilliQ water (EMD Millipore, Burlington, MA, USA) was used.

***In vitro phantom preparation****.* Our first phantom was prepared to measure the CEST MR contrast produced by aspirin and its metabolites in buffer. This phantom was prepared by dissolving SA, salicyl acyl glucuronide (SAG), salicylurate (SU), or 2,5-dihydroxybenzoic acid (2,5-DHB) at 10 mM in phosphate buffered saline (PBS) and titration to pH values of 6.3, 6.6, 6.9 and 7.2, respectively. A second phantom was prepared to test the impact of albumin and other blood proteins on the generated CEST MRI contrast. Two sets of SA tubes were prepared with one set prepared by dissolving 5 mM sodium salicylate and 25% lyophilized human blood serum (Seronorm^TM^, Sero, Billingstad, Norway) in PBS. The second set was prepared using 5 mM sodium salicylate solution in PBS and adding lactate, glucose, and calcium to match the concentrations of these in 25% human blood serum. The solutions were then titrated to pH values of 6.0, 6.3, 6.6, 6.9, 7.2 and 7.5 using 0.5-1 M hydrogen chloride and sodium hydroxide solutions.

***Cell experiments*.** We used four human breast cancer cell lines, MDA-MB-231 (ATCC Cat# HTB-26, RRID:CVCL_0062), SUM159 (RRID:CVCL_5423), SKBR3 (ATCC Cat# HTB-30, RRID:CVCL_0033), and MDA-MB-468 (ATCC Cat# HTB-132), which were obtained from the American Type Culture Collection (ATCC, MD, USA). These cell lines tested negative for mycoplasma using a PCR-based MycoDtect kit (Greiner Bio-One, Monroe, NC) and were authenticated by STR profiling. MDA-MB-231 cells were cultured in RPMI-1640 medium (Invitrogen, Thermo Fisher Scientific, Waltham, MA, USA) supplemented with 10% FBS, while SUM159 cells were cultured in DMEM media supplemented with 10% FBS. SKBR3 cells were cultured in McCoy's 5A medium (ATCC) supplemented with 10% FBS. MDA-MB-468 cells were cultured in DMEM media supplemented with 10% FBS and 1% GlutaMAX (Invitrogen). To perform CEST MRI studies on live cells, ten million cells were trypsinized and resuspended in 200μL PBS with or without 25mM Aspirin DL-lysine for forty minutes at 37^o^C in 1.5 mL Eppendorf tubes before CEST MRI [n=3 each, per cell line]. We have compared cell viability before and after the 40 min incubation using a standard cell viability assay with 3-(4,5-dimethylthiazol-2-yl)-2,5-diphenyl-2H-tetrazolium bromide (MTT) and found the cell viability to be 95% on average. Cells were used for no more than 10 passages after thawing.

***Animal studies.*** All mouse experiments were performed in accordance with protocols approved by the Johns Hopkins University Institutional Animal Care and Use Committee (IACUC). Male C57BL/6 mice of age 4-6 weeks and weighing 20-25g (Jackson Laboratory, Bar Harbor, ME, USA) were maintained under specific pathogen free conditions in the Johns Hopkins University animal facility. For healthy control studies, 2 groups of healthy mice [n= 3 for SA and n= 6 for aspirin] were prepared for imaging by placing a catheter into the tail vein for administering contrast agents, i.e., SA or injectable aspirin (aspirin DL-lysine) during CEST MRI acquisition for monitoring contrast agent uptake in kidneys. For tumor bearing mouse studies, two million triple-negative human MDA-MB-231 breast cancer cells or two million triple-negative human SUM159 breast cancer cells were inoculated into right 4^th^ mammary fat pad of 6-8 weeks old female athymic nude mice (Envigo RMS LLC, Indianopolis, IN, USA). Tumor-bearing mice [n=29, see **Table 1** for experimental groups] with tumor sizes ranging between 300-500 mm^3^ were used to perform CEST MRI experiments by administering 100 µL injectable aspirin ranging from 0 mM to 300 mM.

***PGE2 ELISA.*** Cell pellets from each cell line were harvested and suspended in 25 mM aspirin DL-Lysine dissolved in PBS at a density of five million cells per 100 µL and left at room temperature for 40 minutes. Afterwards, the cell samples were spun down at 13,000 rpm for 5 minutes and the supernatant was collected and diluted for subsequent PGE2 analysis. Each condition was repeated in triplicate. For tissue experiments, approximately 30 mg of ground tumor xenograft tissue from each animal was added to PBS (10 mg/mL w:v), homogenized and left at room temperature for 40 minutes. The samples were then centrifuged as per the cell protocol, with the supernatant collected and diluted 1000-fold before being added to the 96-well ELISA plate included in the Prostaglandin E2 ELISA Kit (ab133055, Abcam, Cambridge, MA, USA). This kit relies on an alkaline phosphatase-conjugated PGE2 antigen and a PGE2-specific antibody. After incubation, para-Nitrophenylphosphate (pNpp) substrate was added, and samples were analyzed using an Epoch microplate spectrophotometer at 405nm.

***Western Blot.*** Cell pellets or tumor xenografts were harvested and suspended in RIPA (Radioimmunoprecipitation) lysis buffer with protease inhibitors (Thermo Fisher Scientific, Waltham, MA). Samples were sonicated over three cycles of five one-second bursts and centrifuged at 13,000 rpm for 15 minutes at 4℃. Supernatants were collected and protein concentration was measured by using a BCA Protein Assay Kit (Thermo Fisher Scientific, Waltham, MA, USA). Protein samples were analyzed by SDS-PAGE and transferred onto PVDF membranes (EMD Millipore, Darmstadt, Germany). The membranes were blocked with 5% non-fat silk milk (EMD Millipore, Darmstadt, Germany) at room temperature for 1 h, then incubated with primary antibodies at 4℃ overnight. On the second day, membranes were incubated with horseradish peroxidase-conjugated secondary antibodies at room temperature for 1 h, then visualized with ChemiDoc MP Imaging System (Bio-Rad, Hercules, CA, USA) following reaction with PierceTM ECL Plus Western Blotting Substrate (Thermo Fisher Scientific, Waltham, MA, USA). Western blot data were processed with Image Lab software version 4.0.1 (Bio-Rad). GAPDH was used as loading control and for normalization. Antibodies and dilutions: anti-COX-1 (ab109025, 1:1000), anti-COX-2 (ab15191, 1:1000) were purchased from Abcam (Cambridge, MA, USA); anti-GAPDH (G8795, 1:3000) was purchased from Sigma-Aldrich (St. Louis, MO, USA); anti-rabbit IgG (7074, 1:2000) and anti-mouse IgG (7076, 1:2000) were purchased from Cell Signaling Technologies (Danvers, MA, USA).

***MR data acquisition.*** MR experiments of phantoms were performed on a Bruker 11.7 T vertical bore spectrometer using a 25 mm transmit/receive volume coil (Bruker BioSpin MRI GmbH, Ettlingen, Germany). All experiments were carried out at 37^0^C. Using 3-plane localizer and axial T_2W_ RARE sequences, a center slice of 1.2 mm thickness was located in the phantom for acquiring CEST MRI data. High resolution T_2W_ images of the center slice were obtained using TE/TR = 30 ms/2.5 s, number of averages = 2, RARE factor = 8, matrix size = 128x128, FOV = 16x17 mm^2^ and spatial resolution = 125x132 μm^2^ respectively. A RARE sequence with CW saturation pulse of 3 sec duration and various RF saturation powers of B_1_ = 1.2, 2.4 3.6, 4.7, 6.0, 7.2, 10.8 and 12 μT was used to generate QUESP data. CEST weighted MRI images were acquired at each RF saturation power by incrementing the saturation frequency every 0.35 ppm from -16 to 16 ppm using centric encoding. Other CEST parameters were TR = 10 s, effective TE = 4.5 ms, RARE Factor = 32, matrix = 64 × 64, FOV = 16x17 mm^2^, spatial resolution = 250x265 μm^2^, slice thickness = 1.2 mm. The total acquisition time for each CEST experiment using one saturation RF power was 30 min 40 sec.

MRI experiments of breast cancer cell suspensions were performed on a Bruker 11.7 T vertical bore spectrometer using a 25 mm transmit/receive volume coil (Bruker BioSpin MRI GmbH, Ettlingen, Germany). All experiments were carried out at 37^0^C. Using 3-plane localizer and axial T_2W_ RARE sequences, a center slice of 1.2 mm thickness was located within the cell suspension for acquiring CEST MRI data. High resolution T_2W_ images of the center slice were obtained using TE/TR = 30 ms/2.5 s, number of averages = 2, RARE factor = 8, matrix size = 128x128, FOV = 16x17 mm^2^ and spatial resolution = 125x132 μm^2^, respectively. A RARE sequence with CW saturation pulse of 3 sec duration and saturation B_1_ = 6.0 μT was used to generate Z-spectra. CEST weighted MRI images were acquired by incrementing the saturation frequency every 0.35 ppm from -16 to 16 ppm using centric encoding. Other CEST parameters were TR = 10 s, effective TE = 4.5 ms, RARE Factor = 32, matrix = 64 × 64, FOV = 16x17 mm^2^, spatial resolution = 250x265 μm^2^, slice thickness = 1.2 mm. The total acquisition time for each CEST experiment using one saturation RF power was 20 mins.

*In vivo* MRI experiments were performed on 11.7 T horizontal bore Bruker Biospec scanner (Bruker Biosciences, Billerica, MA) using an 8-channel mouse body phase array coil. Mice were anesthetized using 0.5-2% isoflurane with the respiration rate continuously monitored and maintained between 30-45 breaths per min. Body temperature was maintained at 37^0^C with a water circulation bed. A 3-plane localizer sequence was used for locating mouse kidneys and mouse position was adjusted to center the tumor in the RF coil. Using T_2W_ RARE sequence, 21-25 contiguous axial slices were acquired and then a center slice of 1.5 mm thickness was selected for CEST MRI of kidneys, while the tumor slice was kept at the center for tumor bearing mice. For 1.5-mm tumor and kidney slices, a 256x256 high resolution T_2W_ RARE image was acquired using TE/TR = 20 ms/4 s, FOV =28x18 mm^2^, number of averages = 1, RARE factor = 8. Shimming was performed on the tumor volume to obtain high B_0_ homogeneity on the tumor. For studies in control mice without tumors, 100 µL of 300 mM (~240mg/kg) SA or aspirin DL-lysine dissolved in deionized (DI) water was administered through the tail vein of each group of mice [n=3]. For tumor bearing mice, 100 µL of 0 mM, 150 mM, 200 mM or 300 mM aspirin DL-lysine were administered [n=29] as indicated in **Table 1.**

CEST-RARE images were acquired to monitor dynamic CEST MRI contrast during injection of aspirin or SA for 70 min duration using a recently developed oversampled two offset protocol that significantly improves CNR 49. RF saturation power was toggled between 6 and 3.6μT for each set of offset values of ±9.6 ppm. 10 sets of M_0_ images (20,000 Hz) at each RF saturation power were acquired at the end of the experiment for CEST signal normalization. CEST saturation was applied for a duration of 3 sec using 10 block pulses of each 300 ms and 10 μs inter-pulse delay. CEST MRI parameters used were TE/TR = 3.49 ms/10 s, number of averages = 1, RARE factor = 32, matrix size = 48x48, FOV = 32x20 mm^2^, spatial resolution = 625x416 μm^2^, centric encoding. The experimental time for each RF power acquisition was 20 sec and CEST data were collected for 1 hour 10 minutes after administering the contrast agent. Pre-injection CEST images were also acquired at two offsets of ±9.6 ppm for two RF saturation powers of 6 and 3.6μT, respectively. For voxel wise spline interpolated B_0_ inhomogeneity correction of CEST weighted images, WASSR-CEST data was acquired using 1.2μT RF saturation for 42 saturation offsets between ±1.5 ppm. The same experimental parameters were used for all salicylate tail vein injections. No CEST MRI experiments were conducted on tumor-bearing mice injected with 0 mM aspirin. Rather, they were left anesthetized for the same duration of time prior to sacrifice. For comparison, gadolinium (Gd) experiments with Gd-HPDO3A (Gadoteridol ProHance, Brocco Diagnostics, Milan, Italy) were conducted on three MDA-MB-231 and three SUM159 tumor-bearing mice. Gd enhanced MRI was performed two days prior to aspirin enhanced CEST MRI to allow sufficient time for clearance of Gd chelates from mice. The dynamic contrast enhanced (DCE) MRI parameters for Gd enhanced MRI were as follows: TE/TR = 1.02 ms/10.4 ms, number of averages = 1, matrix size = 66x96, FOV=28x28mm^2^, spatial resolution= 292x292 μm^2^, thickness=1.2mm. The experimental time for each acquisition was 10 sec for three slices repeated 1000 times, with 200 µL of Gadoteridol (ProHance©, Brocco Diagnostics Inc) injected via the tail vein at T=0 s.

***Data processing.*** CEST MRI data processing was performed using custom written MATLAB scripts (v9.6/R2019a, The MathWorks Inc., Natick, MA). For *in vitro* studies, CEST z-spectra were calculated from the mean of a region of interest (ROI) placed over each tube after performing voxel-based spline interpolated B_0_ correction as described previously 71. For studies on phantoms, CEST signal for each tube was quantified by calculating MTR_asym_ at offsets ±9.6 ppm for SA, ±5.5 and ±1.5 ppm for SAG and ±8.5 ppm for 2,5-DHB. We used the formula of MTR_asym_ = M_z_(-Δω) - M_z_(+Δω)/ M_0_, where M_z_(-Δω), M_z_(+Δω) are the water signal intensities with RF saturation at offsets ±Δω, and M_0_ is the water signal intensity in the absence of saturation pulses. Exchange rate measurements were performed by fitting the QUESP data as described earlier 72 for SA and 2,5-DHB phantoms. For SAG phantom z-spectra and MTR_asym_, this fitting was performed using four pool Bloch McConnell simulations to account for the additional hydroxyl protons in this compound.

For animal studies, z-spectra were extracted from both pre- and post-contrast CEST MR images pixel by pixel. Mean pre-contrast z-spectra were subtracted from all post-contrast z-spectra to proceed with the calculation of dynamic CEST contrast buildup kinetics. Moving time average was applied to the data using the 'smooth' routine in MATLAB to minimize motion induced signal fluctuations. The smoothing factor used in the averaging process was ~20 neighboring images, with this choice based on the size of the data and the range of fluctuations. CEST MRI contrast kinetics were calculated for B_1_= 6μT CEST data of SA and aspirin DL-lysine administration. Average contrast and error bars were calculated for 3-4 mice in each group. After smoothing, 8-10 images were averaged to generate the corresponding dynamic CEST MR contrast images. For calculating the contrast, CEST images at 9.6 ppm were employed and time-course curves were obtained by calculating the average saturation transfer (ST) = [1-M_z_(+Δω)]/M_0_ over pixels selected by two ROIs, one over each kidney or an ROI over the tumor or muscle tissue. Maximum contrast maps were generated by calculating the pixel-by-pixel maximum ST on two ROIs, one over each kidney or over the tumor or muscle tissue, overlaid on the corresponding T_2W_ image as described previously 71. Raw unmasked images are shown in **Figure S12**. The time averaged maximum contrast images were calculated by taking 8-10 images at corresponding post contrast agent administration times. *In vivo* B_0_ inhomogeneity maps were generated from the WASSR experiment performed for each mouse prior to contrast agent injection. WASSR Z-spectra for ROIs drawn over both kidneys or the tumor or muscle were extracted and interpolated in MATLAB using cubic spline interpolation. The water shift was measured pixel by pixel to obtain ΔB_0_ maps over the kidney and tumor and muscle. ΔB_0_ maps were overlaid on the corresponding high resolution T_2W_ images to obtain ΔB_0_ maps (**Figure 3**).

Gadolinium DCE MRI data were processed using custom written MATLAB scripts (v9.6/R2019a, The MathWorks Inc., Natick, MA). Dynamic gadolinium contrast enhanced images were extracted pre- and post-contrast administration. Pre-contrast images and gadolinium-enhanced contrast images at 14 min were used to compare with the post-CEST contrast images at 60 min.

## Supplementary Material

Supplementary methods and figures.

## Figures and Tables

**Table 1:** Number of tumor-bearing mice analyzed for each breast cancer xenograft model at each concentration of aspirin injected.

Breast cancer xenograft model	Concentration of aspirin injected	Count (n)
MDA-MB-231	300 mM	4
	200 mM	6
	150 mM	6
	0 mM	3
SUM159	300 mM	4
	0 mM	4

## Abbreviations

2,5-DHB: 2,5-dihydroxybenzoic acid
CEST: chemical exchange saturation transfer
COX: cyclooxygenase
CW: continuous wave
FOV: field of view
Gd: Gadolinium
HPLC: high-pressure liquid chromatography
MALDI: matrix-assisted laser desorption ionization
MRI: magnetic resonance imaging
MS: mass spectrometer
NSAID: non-steroidal anti-inflammatory drug
PGE2: Prostaglandin E2
RARE: rapid acquisition with relaxation enhancement
RF: radio frequency
ROI: region of interest
SA: salicylic acid
SAG: salicyl acyl glucuronide
SPG: salicyl phenolic glucuronide
SU: salicylurate
TE: echo time
TOF: time-of-flight
TR: repetition time
WASSR: water saturation shift referencing
