# Supplementary material for: Unlabeled aspirin as an activatable theranostic MRI agent for breast cancer
Source: Theranostics. 2022 Jan 24;12(4):1937–51. doi: 10.7150/thno.53147 (PMC8825591; doi:10.7150/thno.53147)
Supplement: Supplementary file 1 — Supplementary methods and figures. [file thnov12p1937s1.pdf]

Supplementary Information

Unlabeled Aspirin as an Activatable Theranostic MRI Agent for Breast Cancer

Kowsalya Devi Pavuluri[1], Ethan Yang[2], Vinay Ayyappan[2], Kanchan Sonkar[2], Zheqiong Tan[2], Caitlin M. Tressler[2], Shaowei Bo[1], Adnan Bibic[4], Kristine Glunde[2,3,4]* and Michael T McMahon[1,5]*

[1]Division of MR Research, [2]Division of Cancer Imaging Research, The Russell H. Morgan Department of Radiology and Radiological Science, [3]The Department of Biological Chemistry, [4]Sidney Kimmel Comprehensive Cancer Center, The Johns Hopkins University School of Medicine, Baltimore, MD.
[5]F.M. Kirby Research Center for Functional Brain Imaging, Kennedy Krieger Institute, Baltimore, MD.

* To whom correspondence should be addressed: kglunde1@jhmi.edu, mcmahon@kennedykrieger.org

Keywords: CEST, aspirin, MRI, breast cancer, MALDI, Gadolinium

Contents

Supplementary Tables 3

Supplementary Table 1: Measured CEST properties for the metabolites in the aspirin pathway including intramolecular hydrogen bonded exchangeable protons. 3

Supplementary Figures 4

Supplementary Figure 1. CEST contrast of salicylic acid (SA) and three of its metabolites, salicylurate (SU), salicyl acyl glucuronide (SAG), and 2,5-dihydroxybenzoic acid (2,5-DHB) at pH 6.3, 6.6, 6.9 and 7.2. 4

Supplementary Figure 2. Comparison of CEST properties of salicylic acid (SA) in PBS *versus* blood serum. 5

Supplementary Figure 3. An example of T_{2W}, pre- and post-administration CEST contrast images without masking. 6

Supplementary Figure 4. CEST MRI detection of four SUM159 tumor bearing mice treated with 300 mM of DL-Lys Aspirin 7

Supplementary Figure 5. CEST MRI detection of four MDA-MB-231 tumor bearing mice treated with 300 mM of DL-Lys Aspirin. 8

Supplementary Figure 6. High resolution dynamic contrast enhanced (DCE) MRI images post gadolinium injection compared with CEST images post aspirin injection. 9

Supplementary Figure 7. COX-1 and COX-2 expression levels of tumor xenografts with and without aspirin treatment. 10

Supplementary Figure 8. CEST MRI detection of six MDA-MB-231 tumor bearing mice treated with 200 mM of DL-Lys Aspirin. 11

Supplementary Figure 9. CEST MRI detection of six MDA-MB-231 tumor bearing mice treated with 150 mM of DL-Lys Aspirin. 12

Supplementary Figure 10. MDA-MB-231 tumor xenograft PGE2 concentrations following aspirin injection. 13

Supplementary Figure 11. Average MALDI-TOF mass spectra of SA and 2,5-DHB from treated and untreated SUM159 tumor xenografts. 14

Supplementary Figure 12. Representative ΔB0 15

Supplemental Methods 16

MALDI-TOF mass spectrometry (MS) acquisition method 16

Supplementary Tables

Supplementary Table 1: Measured CEST properties for the metabolites in the aspirin pathway including intramolecular hydrogen bonded exchangeable protons. Based on this table and Fig. 1D, the free -OH group in SA produces CEST contrast, while SAG and SU do not display CEST MR contrast at ~9.6 ppm.

	Salicylic Acid (SA)[a]	Salicylic Acid Acyl-β-D-glucuronide (SAG)		Salicylurate (SU) [b]	2,5 Dihydroxy benzoic acid (2,5-DHB)[a]
Δω (ppm from water)	9.6	5.5	1.5	-	8.5
K_{sw} (s^{-1})<br>pH 6.3<br>6.6<br>6.9<br>7.2	 4,503±70<br>3,083±30<br>2,597±40<br>1,999±30	 7,500±600<br>9,000±800<br>10,500±900<br>10,500±900	 4,400±600<br>4,200±300<br>3,700±400<br>1,900±400	 -<br>-<br>-<br>-	 2,548±60<br>1,943±50<br>968±10<br>601±20
Concentration (mM)	20	20		20	20
Contrast (%) for pH 6.6 at 6 μT RF saturation power	11.9	4.7	21	0	14.2

[a]As measured in Cassidy, M. C., Chan, H. R., Ross, B. D., Bhattacharya, P. K. & Marcus, C. M. In vivo magnetic resonance imaging of hyperpolarized silicon particles. *Nature Nanotechnology* **8**, 363-368, doi:10.1038/nnano.2013.65 (2013)

[b]CEST contrast not detected for this metabolite

Supplementary Figures

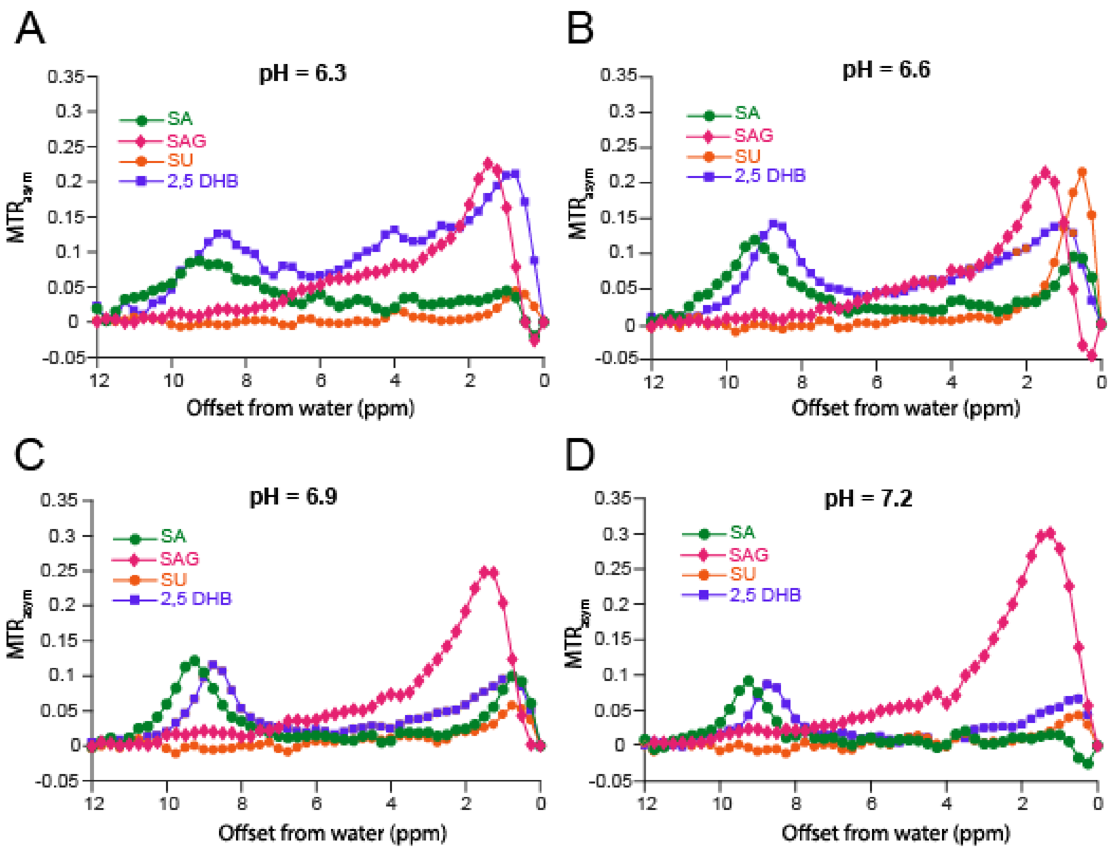

Supplementary Figure 1. CEST contrast of salicylic acid (SA) and three of its metabolites, salicylurate (SU), salicyl acyl glucuronide (SAG), and 2,5-dihydroxybenzoic acid (2,5-DHB) at pH 6.3, 6.6, 6.9 and 7.2. Overall, SA retained similar levels of MTR_{sym} contrast at all pH values, while SAG signal improved with increasing pH level. SU had the highest contrast at pH 6.6. 2,5-DHB contrast at 8.5 ppm did not change with pH but lost its secondary contrast at 1.5 ppm with increasing pH.

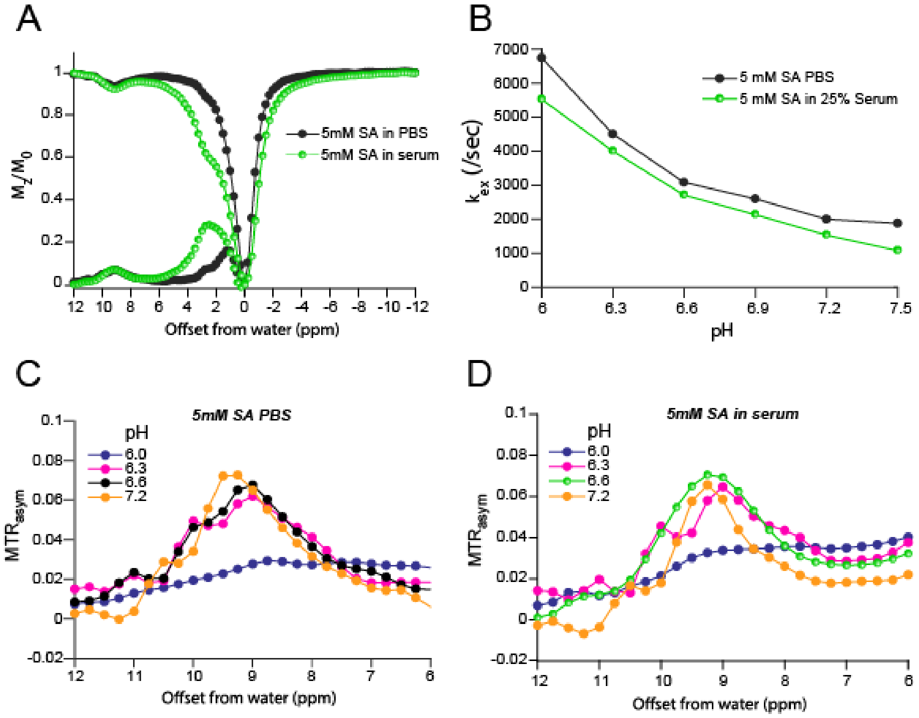

Supplementary Figure 2. Comparison of CEST properties of salicylic acid (SA) in PBS *versus* blood serum. (A) Z-spectra showing SA contrast in PBS and serum. **(B)** CEST exchange rate of SA in PBS and serum. MTR_{asym} of SA **(C)** in PBS and **(D)** in serum from pH 6.0 to 7.2. No major differences in SA CEST properties were observed, indicating that the free -OH group in SA retained its contrast in blood serum.

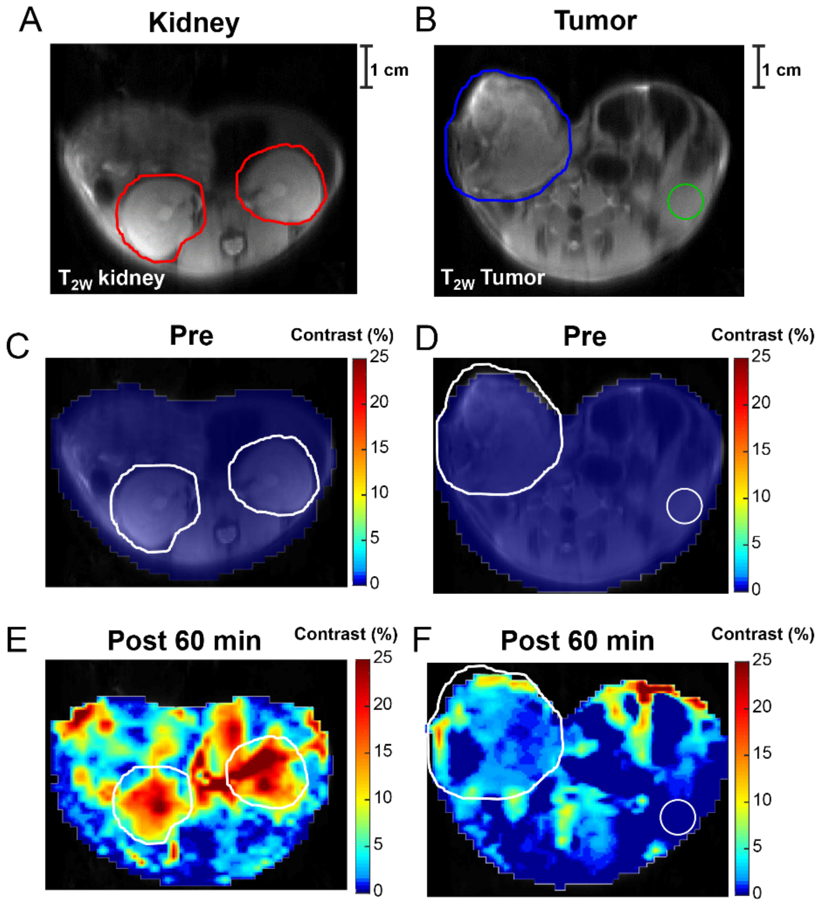

Supplementary Figure 3. An example of T_{2W}, pre- and post-administration CEST contrast images without masking. (A), **(B)** T_{2W} images of a representative mouse with kidney (red line), tumor (blue line) and muscle (green line) regions indicated. **(C)**, **(D)** Pre-CEST MRI contrast images of kidney and tumor and muscle with no mask. **(E)**, **(F)** Post-60 min CEST MRI contrast images with no mask, with kidney, tumor and muscle regions identified. Kidney has significantly higher CEST MRI contrast compared to the tumor.

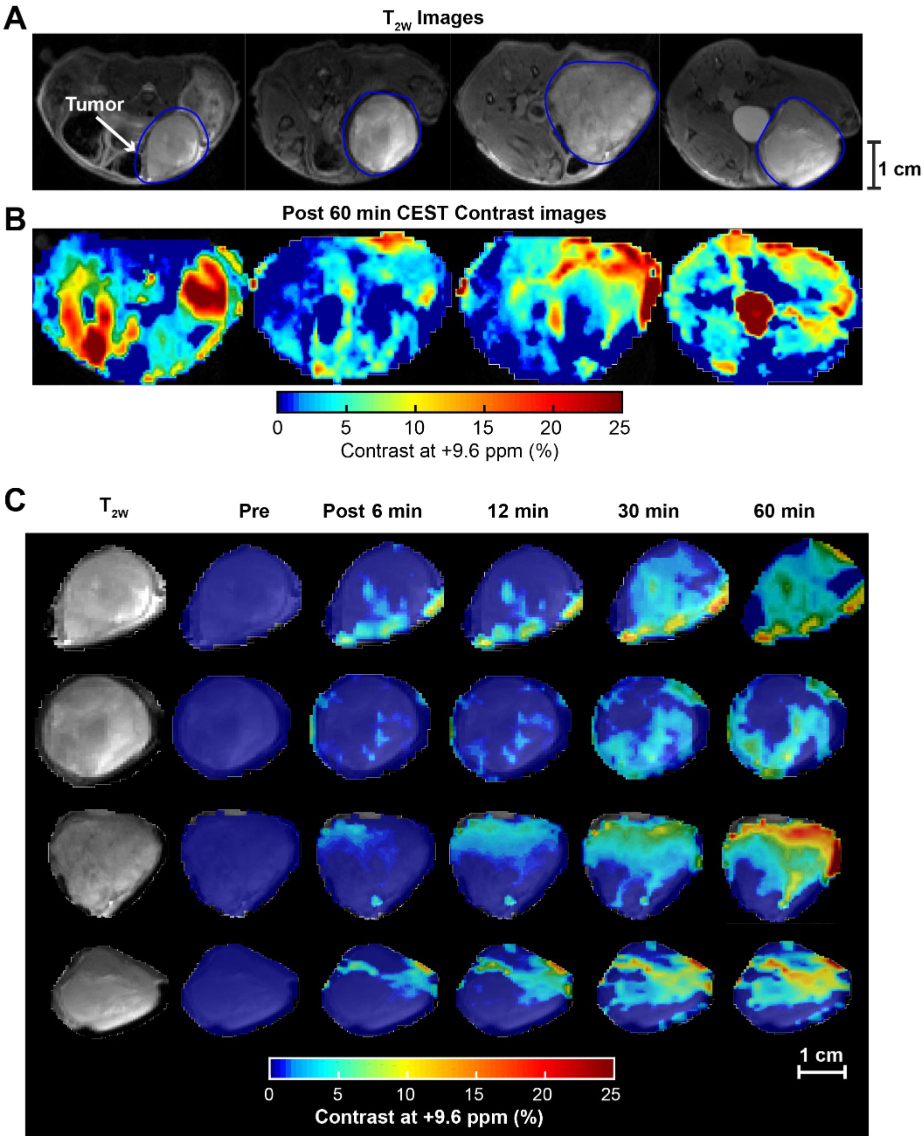

Supplementary Figure 4. CEST MRI detection of four SUM159 tumor bearing mice treated with 300 mM of DL-Lys Aspirin. (A) High resolution T_{2w} images with the tumor region outlined in blue. **(B)** CEST MR contrast images at 9.6 ppm of the entire cross section at 60 mins after injection. **(C)** High resolution T_{2w} image and pre- and post-injection dynamic CEST MR contrast images of the tumor regions overlaid on T_{2w} image over the course of 60 mins, with the average CEST MRI contrast at 9.6 ppm at each time point as indicated.

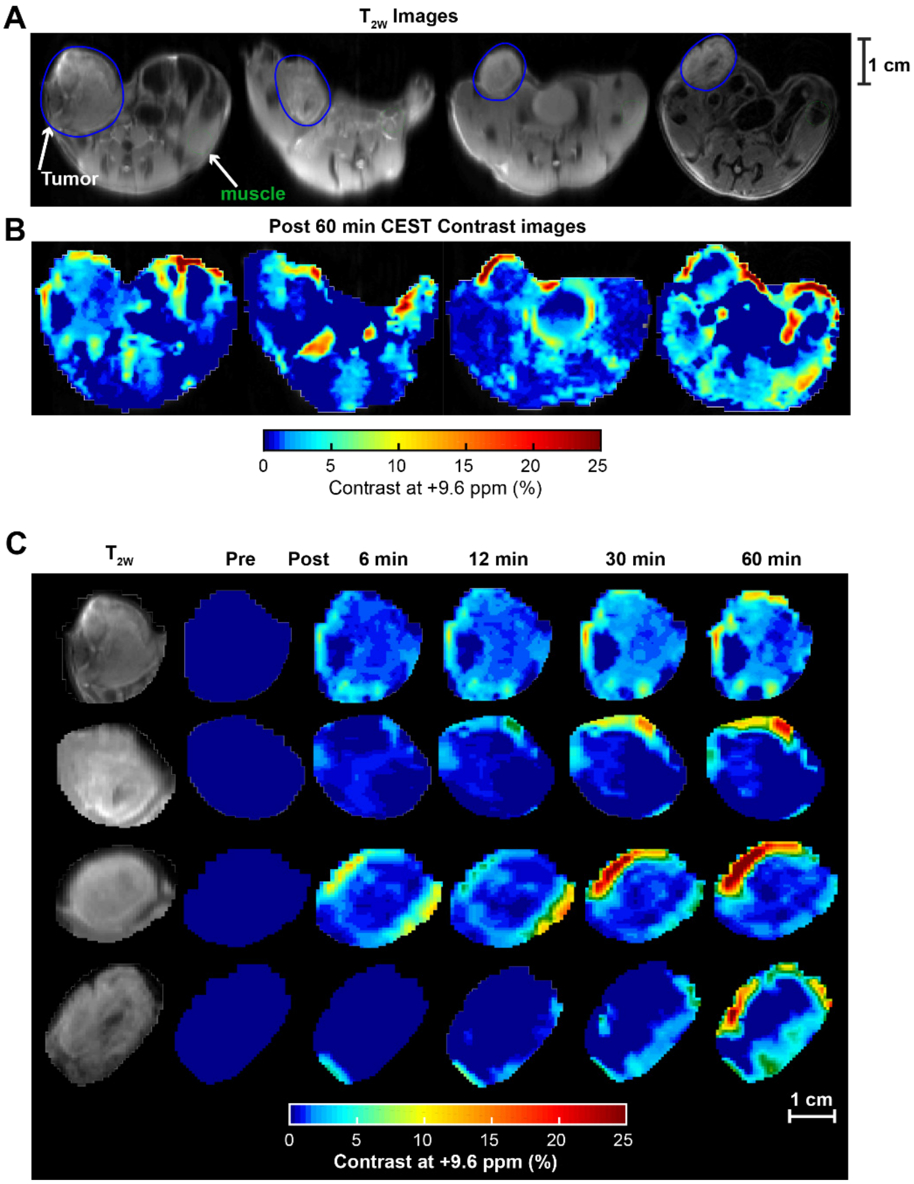

Supplementary Figure 5. CEST MRI detection of four MDA-MB-231 tumor bearing mice treated with 300 mM of DL-Lys Aspirin. (A) High resolution T_{2w} images with the tumor region outlined in blue. **(B)** CEST MR contrast images at 9.6 ppm of the entire cross section at 60 mins after injection. **(C)** High resolution T_{2w} image and pre- and post-injection dynamic CEST MR contrast images of the tumor regions overlaid on T_{2w} over the course of 60 mins, with the average CEST MRI contrast at 9.6 ppm at each time point as indicated.

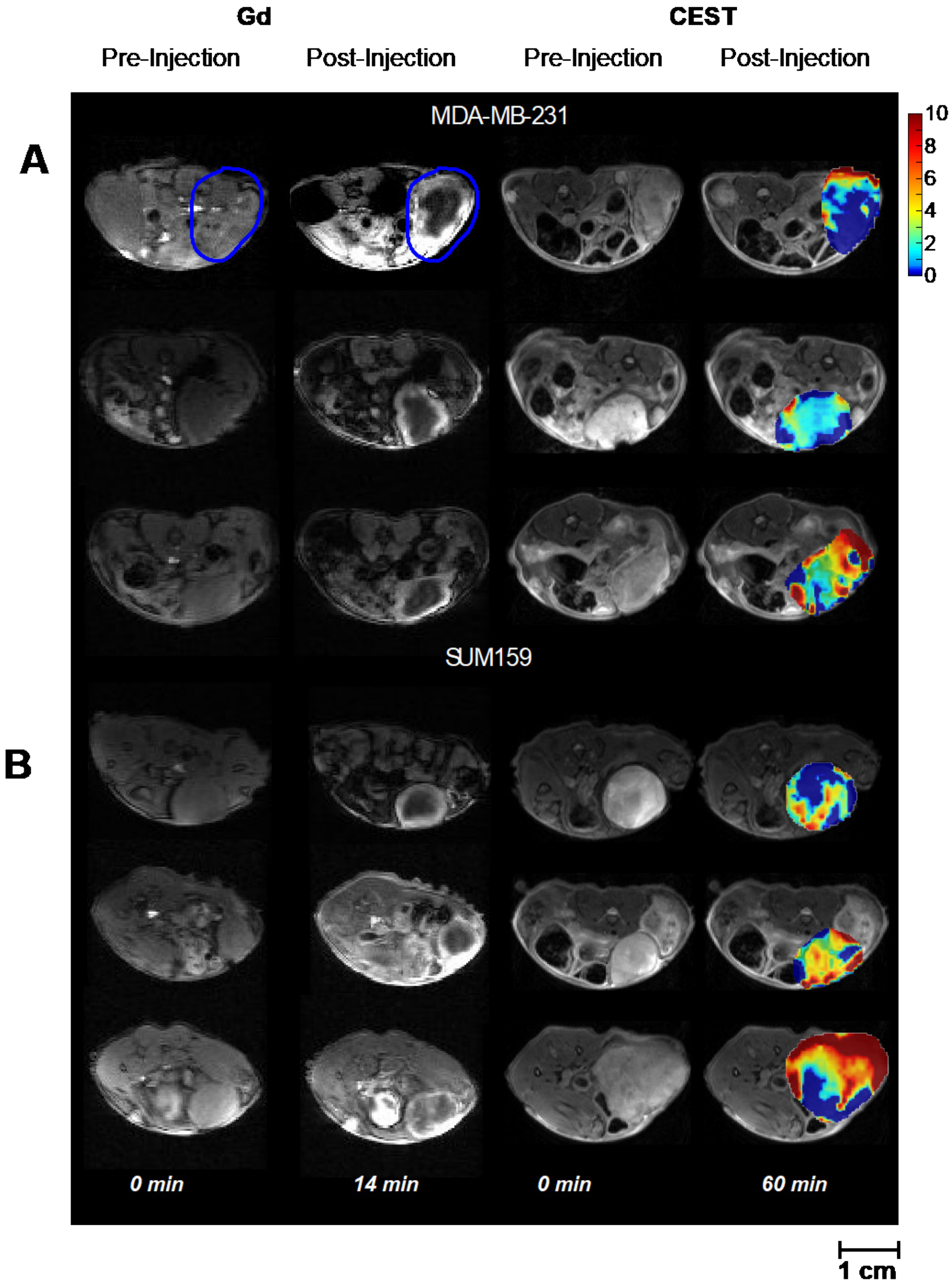

Supplementary Figure 6. High resolution dynamic contrast enhanced (DCE) MRI images post gadolinium (Gd) injection compared with CEST images post aspirin injection. (A) Three MDA-MB-231 tumor bearing mice and **(B)** three SUM159 tumor bearing mice were randomly selected for this analysis. After 14 mins of injection with Gd-HPDO3A (Gadoteridol or ProHance®, Gd), the tumor region is clearly visible. SA CEST MRI contrast of the same tumor acquired 48 hours later at 60 min post-injection of 200 mM aspirin for MDA-MB-231 and 300 mM for SUM159 clearly showed tumor enhancement as well. Positioning and imaging slice placement were done independently for Gd-enhanced MRI and SA CEST MRI measurements with two days in between to allow for complete clearance of Gd prior to SA CEST MRI. Therefore, some tumor shapes and sizes look different for the same tumors because of variations in positioning and placement of the imaging slice for Gd-enhanced MRI *versus* SA CEST MRI.

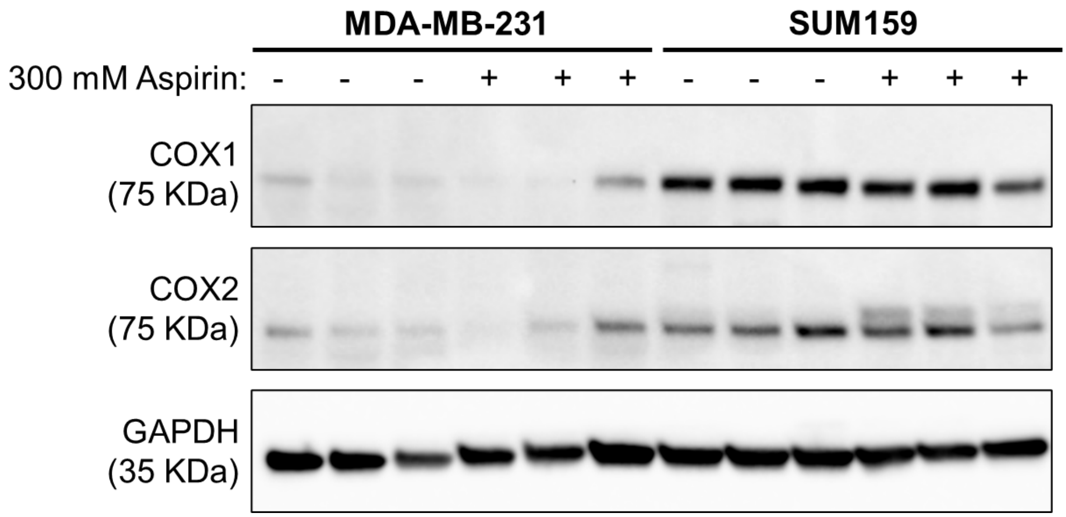

Supplementary Figure 7. COX-1 and COX-2 expression levels of tumor xenografts with and without aspirin treatment. Western blot analysis of MDA-MB-231 and SUM159 tumor xenografts with and without 300 mM aspirin treatment (n=3 for each condition). Compared to MDA-MB-231, SUM159 xenografts presented much higher protein expression levels of both COX-1 and COX-2. Aspirin treatment did not affect the COX-1 and COX-2 expression level in either tumor xenograft model. Protein extracts of MDA-MB-231 and SUM159 tumor xenografts were loaded into each lane. Sodium dodecyl sulphate–polyacrylamide gel electrophoresis (SDS-PAGE) was performed using a 7.5% resolving gel and transferred onto polyvinylidene difluoride (PVDF) membranes for blotting with anti-COX1 and anti-COX2. Glyceraldehyde 3-phosphate dehydrogenase (GAPDH) was used as loading control.

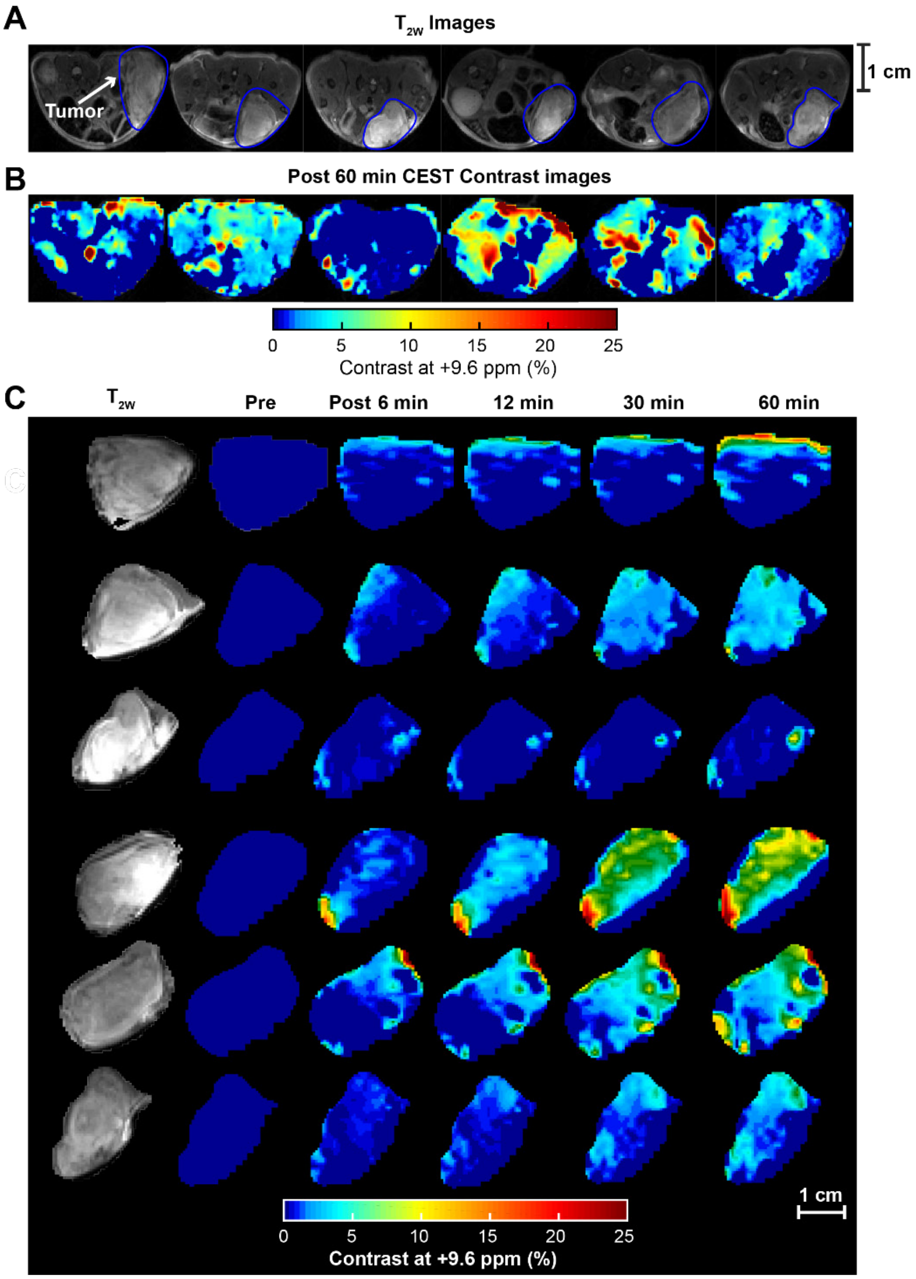

Supplementary Figure 8. CEST MRI detection of six MDA-MB-231 tumor bearing mice treated with 200 mM of DL-Lys Aspirin. (A) High resolution T_{2w} images with the tumor region outlined in blue. **(B)** CEST MR contrast images at 9.6 ppm of the entire cross section at 60 mins after injection. **(C)** High resolution T_{2w} image and pre- and post-injection dynamic CEST MR contrast images of the tumor regions overlaid on T_{2w} over the course of 60 mins, with the average CEST MRI contrast at 9.6 ppm at each time point as indicated.

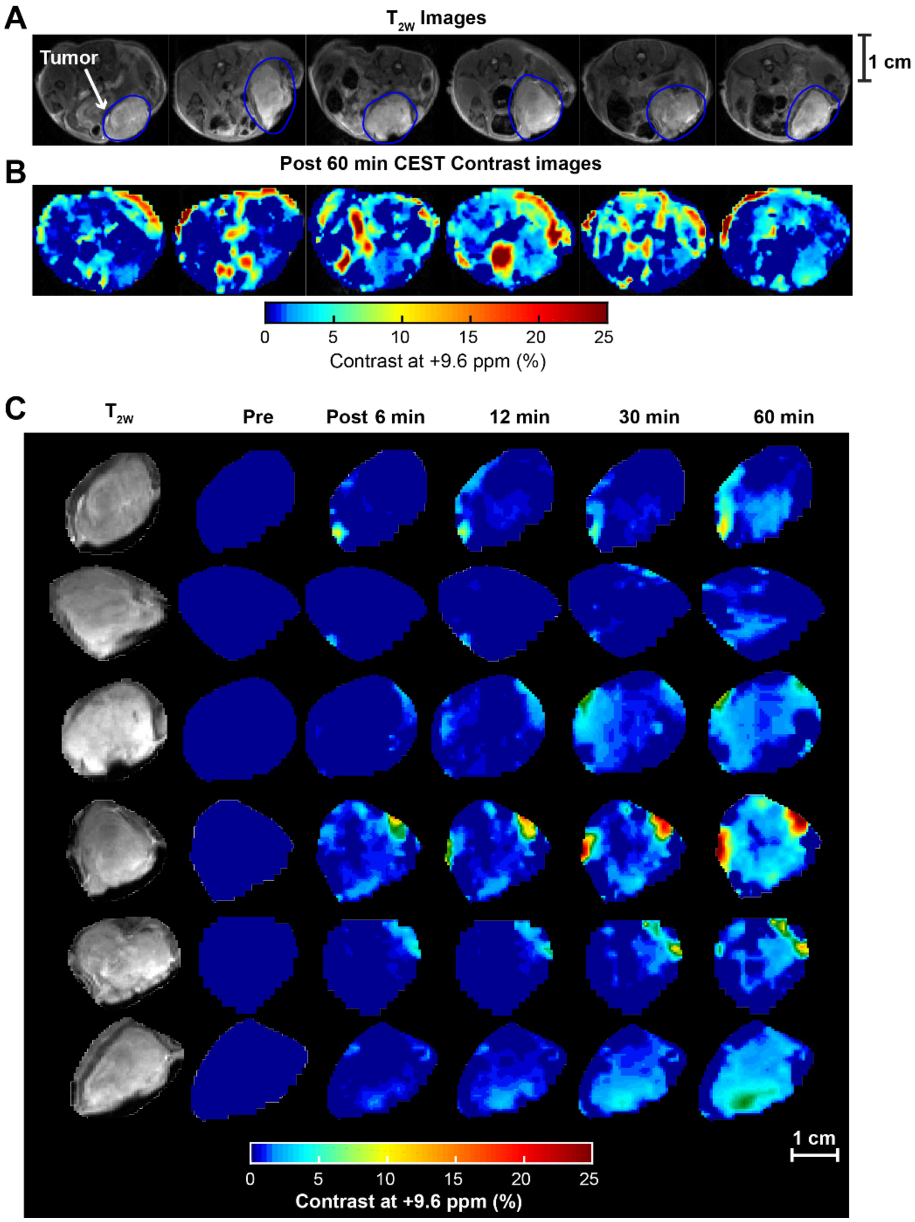

Supplementary Figure 9. CEST MRI detection of six MDA-MB-231 tumor bearing mice treated with 150 mM of DL-Lys Aspirin. (A) High resolution T_{2w} images with the tumor region outlined in blue. **(B)** CEST MR contrast images at 9.6 ppm of the entire cross section at 60 mins after injection. **(C)** High resolution T_{2w} image and pre- and post-injection dynamic CEST MR contrast images of the tumor regions overlaid on T_{2w} over the course of 60 mins, with the average CEST MRI contrast at 9.6 ppm at each time point as indicated.

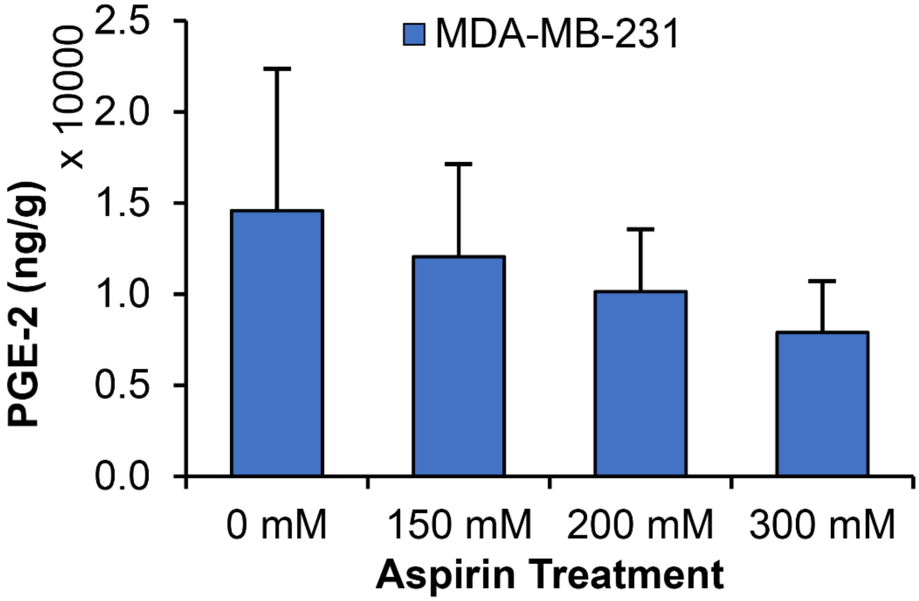

Supplementary Figure 10. MDA-MB-231 tumor xenograft PGE2 concentrations following aspirin injection. On average, the concentration of PGE2 decreased from 14,800 ng/g to 7,800 ng/g as the concentration of aspirin treatment increased from 0 mM to 300 mM, supporting the irreversible inactivation of COX-1/-2 enzymes by aspirin. Data shown are mean ± standard deviation.

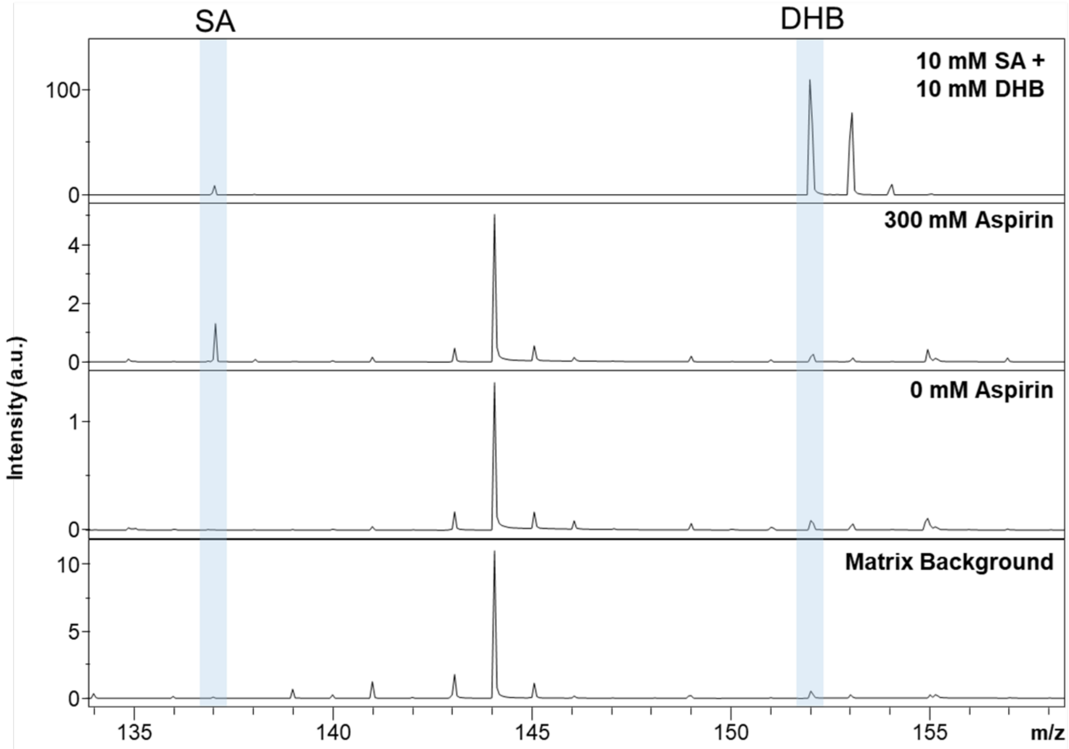

Supplementary Figure 11. Average MALDI-TOF mass spectra of SA and 2,5-DHB from treated and untreated SUM159 tumor xenografts. From top to bottom: standard mix containing 10 mM SA and 10 mM 2,5-DHB; average mass spectrum from SUM159 tumor xenograft treated with 300 mM aspirin; average mass spectrum from control SUM159 tumor xenograft treated with vehicle control (0 mM aspirin); MALDI matrix background spectrum. MALDI-TOF mass spectrometry (MS) profiling results confirm that the major CEST MR signals detected in breast tumor models are indeed from SA and not 2,5-DHB, evident from high SA peak and no DHB signal in tissue sections from SUM159 tumors treated with 300 mM aspirin. No SA peak was detected in the control SUM159 tumor xenograft. Data was acquired in negative ion mode, with norharmane as the matrix. Experimental details are provided in Supplemental Methods.

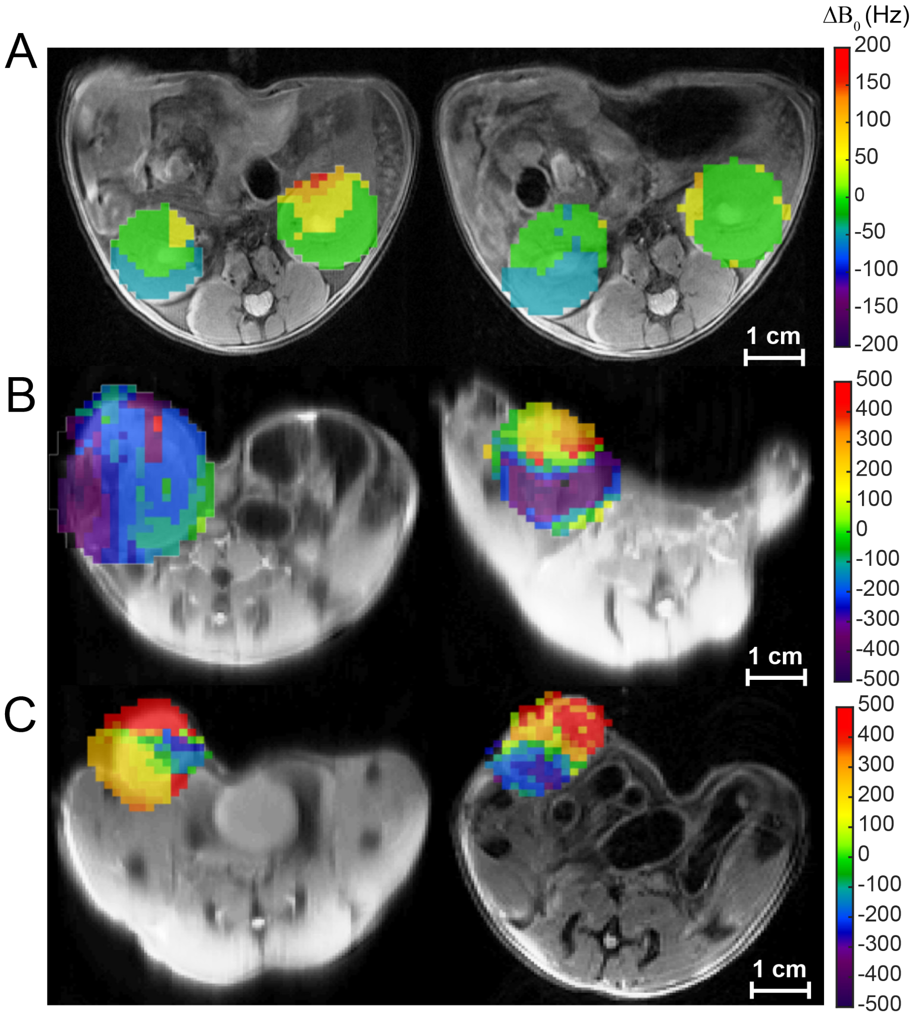

Supplementary Figure 12. Representative ΔB0 maps for kidney and tumor. ΔB0 maps are generated using WASSR experiment performed using 0.5 μT RF saturation power and 42 offsets between +1.5 ppm and -1.5 ppm. (A) Kidney ΔB_0 maps with average ΔB_0 of -14.8 and 14.5 Hz over both kidneys, respectively. **(B)**, **(C)** ΔB_0 maps for four MDA-MB-231 breast tumor-bearing mice where average ΔB_0 over the tumor are of -225.6, -386.2, 435.7, 171.3 Hz, respectively.

Supplemental Methods

MALDI-TOF mass spectrometry (MS) acquisition method

Standard solutions of salicylic acid (SA) and 2,5-dihydroxybenzoic acid (2,5-DHB) at 20 mM were prepared in MilliQ water. An equal volume of each standard solution was mixed to prepare a standard mix of SA and 2,5-DHB at 10 mM each. For on-tissue metabolite detection, one fresh frozen SUM159 tumor xenograft from a mouse treated with 300 mM injectable aspirin DL-lysine and one SUM159 tumor xenograft from a mouse treated with control solution (0 mM, untreated) were randomly chosen.

On an indium-tin-oxide (ITO) coated glass slide, one 0.5 µL drop of the standard mix (10 mM of salicylic acid + 10 mM of 2,5 dihydroxybenzoic acid) was deposited on the left side of the slide, while one 10 µm section each of the treated and untreated SUM159 xenografts were cryosectioned (Leica CM-1860-UV) and thaw-mounted on the right side of the slide. After desiccation for ~30 mins, norharmane (10 mg/mL in 70% MeOH) was spray-coated onto the entire slide with an HTX M5 Sprayer (HTX Technologies, Chapel Hill, NC) as per the following protocol: 4 passes, 0.1 mL/min flow rate, criss cross (CC) pattern, 3 mm track spacing, 10 psi, 2L/min gas flow rate, 0s drying time, 40 mm nozzle height.

MS profiling data was acquired on a Bruker rapifleX (Bruker Daltonics, Billerica, Germany) in negative ion reflectron mode with flexControl at 200 shots per spectrum from *m/z* 0-500. Representative spectra were obtained from the standard mix, while a series of spectra were acquired randomly throughout the tissue sections and averaged. Spectral profile of the matrix background was also acquired for comparison.
